# A gut-derived *Streptococcus salivarius* produces the novel nisin variant designated nisin G and inhibits *Fusobacterium nucleatum* in a model of the human distal colon microbiome

**DOI:** 10.1128/mbio.01573-24

**Published:** 2024-12-18

**Authors:** Garreth W. Lawrence, Enriqueta Garcia-Gutierrez, A. Kate O’Mahony, Calum J. Walsh, Paula M. O'Connor, Máire Begley, Caitriona M. Guinane, Paul D. Cotter

**Affiliations:** 1 Department of Biological Sciences, Munster Technological University, Cork, Ireland; 2 Teagasc Food Research Centre, Cork, Ireland; 3 APC Microbiome Ireland, Cork, Ireland; 4 VistaMilk SFI Research Centre, Cork, Ireland; University of Nebraska-Lincoln, Lincoln, Nebraska, USA

**Keywords:** nisin, bacteriocin, antimicrobial, colorectal cancer, *Streptococcus salivarius*, *Fusobacterium nucleatum*, *ex vivo* colon model

## Abstract

*Fusobacterium nucleatum* is a human pathogen associated with intestinal conditions including colorectal cancer. Screening for gut-derived strains that exhibit anti-*F*. *nucleatum* activity *in vitro* revealed *Streptococcus salivarius* DPC6487 as a strain of interest. Whole-genome sequencing of *S. salivarius* DPC6487 identified a nisin operon with a novel structural variant designated nisin G. The structural nisin G peptide differs from the prototypical nisin A with respect to seven amino acids (Ile4Tyr, Ala15Val, Gly18Ala, Asn20His, Met21Leu, His27Asn, and His31Ile), including differences that have not previously been associated with a natural nisin variant. The nisin G gene cluster consists of *nsgGEFABTCPRK* with transposases encoded between the nisin G structural gene (*nsgA*) and *nsgF*, notably lacking an equivalent to the *nisI* immunity determinant. *S. salivarius* DPC6487 exhibited a narrower spectrum of activity *in vitro* compared to the nisin A-producing *Lactococcus lactis* NZ9700. Nisin G-producing *S. salivarius* DPC6487 demonstrated the ability to control *F. nucleatum* DSM15643 in an *ex vivo* model colonic environment while exerting minimal impact on the surrounding microbiota. The production of this bacteriocin by a gut-derived *S. salivarius*, its narrow-spectrum activity, and its anti-*F. nucleatum* activity in a model colonic environment indicates that this strain merits further attention with a view to harnessing its probiotic potential.

**IMPORTANCE:**

*Fusobacterium nucleatum* is a human pathogen associated with intestinal conditions, including colorectal cancer, making it a potentially important therapeutic target. Bacteriocin-producing probiotic bacteria demonstrate the potential to target disease-associated taxa *in situ* in the gut. A gut-derived strain *Streptococcus salivarius* DPC6487 was found to demonstrate anti-*F*. *nucleatum* activity, which was attributable to a gene encoding a novel nisin variant designated nisin G. Nisin G-producing *S. salivarius* DPC6487 demonstrated the ability to control an infection of *F. nucleatum* in a simulated model of the human distal colon while exerting minimal impact on the surrounding microbiota. Here, we describe this nisin variant produced by *S. salivarius*, a species that is frequently a focus for probiotic development. The production of nisin G by a gut-derived *S. salivarius*, its narrow-spectrum activity against *F. nucleatum*, and its anti-*F*. *nucleatum* activity in a model colonic environment warrants further research to determine its probiotic-related applications.

## INTRODUCTION

The human gut microbiome comprises trillions of microbes, some members of which coexist while others compete for essential resources that determine their survival (1). Co-evolution and competition have resulted in the development of several mechanisms that aid in the survival of microorganisms, including the secretion of antimicrobial peptides (2–4). For this reason, it is not surprising that the gut microbiome is regarded as a reservoir of antimicrobial peptides such as bacteriocins that hold therapeutic potential (5–10). Bacteriocins are ribosomally synthesized antimicrobial peptides/proteins that display a narrow- or broad-spectrum of activity (10, 11). As antibiotic-resistant pathogens continue to emerge, bacteriocins have increasingly been studied as potential antimicrobial alternatives to traditional antibiotics (12). Furthermore, bacteriocin-producing probiotic bacteria have the potential to target disease-associated taxa *in situ* in the gut (13).

The genus *Streptococcus* is well-known for its bacteriocin-producing potential, including the production of lantibiotics (14). The prototypical lantibiotic is nisin. First discovered in *Lactococcus lactis* in 1928, variants of the broad-spectrum lantibiotic nisin A have since been reported to be produced by *Streptococcus* species, i.e., strains of *Streptococcus uberis* produce nisin variants nisin U and U2 (15), nisin H is produced by a strain of *Streptococcus hyointestinalis* (16), while nisin *P* is produced by a strain of *Streptococcus agalactiae* (5). More recently, nisin S was reported to be produced by a strain of *Ligilactobacillus salivarius* (17) and nisin E by multiple *Streptococcus equinus* strains (18). Interestingly, a nisin-like peptide, salivaricin D, is produced by a strain of *Streptococcus salivarius*. However, the structure of salivaricin D may form four lanthionine rings in contrast to nisin A which forms five rings (19). Nisin exerts its antimicrobial activity through pore formation and inhibition of cell wall biosynthesis (20). Nisin A was approved as a food preservative in 1953 (21), and in 1988, the FDA granted nisin as generally regarded as safe status. Furthermore, nisin has been investigated to assess its biotherapeutic potential, including the targeting of bacterial pathogens associated with cancer (22).

Several strains of *S. salivarius* produce the lantibiotic salivaricin A, with five variants having been identified to date (23). Notably, *S. salivarius* strain K12, a co-producer of salivaricin B and salivaricin A2 with antagonistic activity against the pathogen *Streptococcus pyogenes,* has been developed as a commercial probiotic and has passed rigorous safety assessment for human use (24, 25). As strains of *S. salivarius* have been shown to benefit human health in numerous clinical trials (26–28), there is considerable merit in screening further strains for traits with a view to the further development of novel probiotics with antimicrobial activity (29).

A potential target for these novel antimicrobial-producing probiotics is the emerging human gram-negative pathogen *Fusobacterium nucleatum*, within the phylum Fusobacteriota, which has been shown to be associated with colorectal cancer (CRC). Higher abundances of *Fusobacterium* and *F. nucleatum* have been identified in the fecal, tissue, and mucosal samples of patients with CRC, relative to the corresponding samples from healthy controls (30–36), and increased abundances of *F. nucleatum* have been found to correlate with poor patient prognosis (37). Evidence suggests that *F. nucleatum* contributes to the development of CRC (38) and thus represents a potential therapeutic target (13). Recent efforts demonstrated that a salivaricin-producing *S. salivarius* strain, DPC6993, suppresses the growth of *F. nucleatum* in an *ex vivo* model of the human colon (39), supporting the potential to use other bacteriocin-producing *S. salivarius* strains to target *F. nucleatum*.

In this study, the intestinal bacterium *S. salivarius* DPC6487, previously isolated from a neonatal fecal sample (6), demonstrated antimicrobial activity against strains of *F. nucleatum*. Further analysis suggests that this strain exhibits a narrow spectrum of antimicrobial activity. Genome sequencing revealed a gene cluster encoding a novel nisin variant, designated nisin G. We analyzed this novel gene cluster and assessed the antimicrobial spectrum of *S. salivarius* DPC6487 compared to a nisin A producer. Additionally, we studied the impact of *S. salivarius* DPC6487 on *F. nucleatum* and the surrounding microbiota in a complex *ex vivo* model of the distal human colon to assess its behavior as a potential therapeutic agent.

## RESULTS

### Characterization of an unknown antimicrobial produced by *S. salivarius* DPC6487

**Fig 1:**
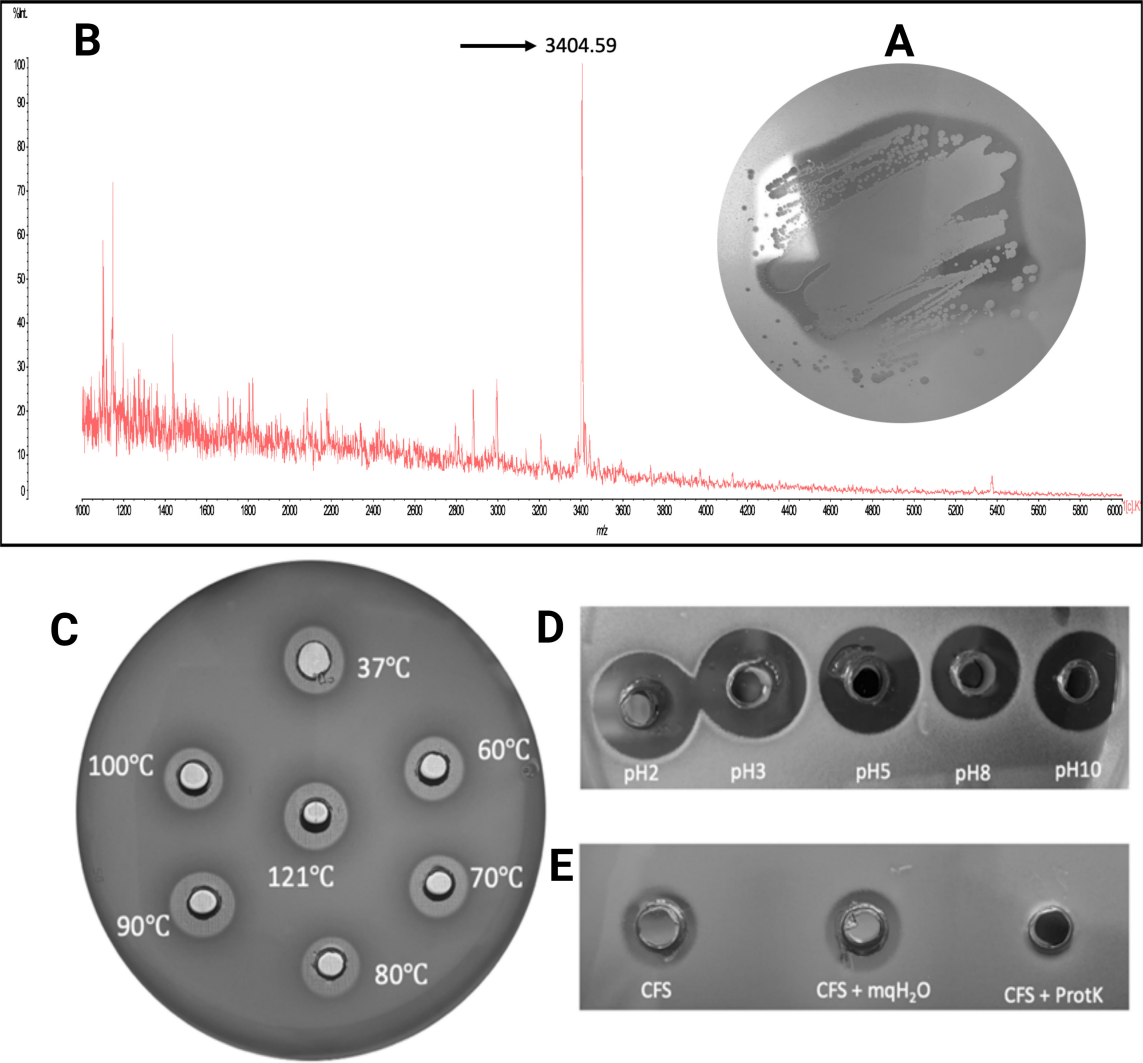
An unknown antimicrobial is produced by *S. salivarius* DPC6487. Deferred antagonism assay whereby *S. salivarius* DPC6487 demonstrated antimicrobial activity against *F. nucleatum* DSM15643 (**A**). The presence of a 3404.59 Da mass was revealed by MALDI-TOF MS (**B**). The antimicrobial activity of *S. salivarius* DPC6487 CFS was assessed against *Lactobacillus delbrueckii* subsp. *bulgaricus* DPC5383 after subjection to heat (**C**), pH (**D**), and proteinase K (**E**).

*S. salivarius* DPC6487 was previously isolated from a neonatal fecal sample in a screening study of the human gut microbiota and was previously reported to demonstrate antimicrobial activity against a strain of *Lactobacillus delbrueckii* subsp. *bulgaricus* (6). In this study*,* further analysis of *S. salivarius* DPC6487 revealed that it demonstrates antimicrobial activity against the pathogen *F. nucleatum* DSM15643 (Fig. 1A). MALDI-TOF colony mass spectrometry of *S. salivarius* DPC6487 showed the presence of a molecule with a mass of 3,405 Da (Fig. 1B).

The antimicrobial activity of cell-free supernatant (CFS) from an overnight culture of *S. salivarius* DPC6487 remained stable after treatment at 37°C, 60°C, 70°C, 80°C, 90°C, 100°C, and 121°C for 10 minutes (Fig. 1C). The activity of the CFS also remained active at pH 2, 3, 5, 8, and 10, with antimicrobial activity being greatest at pH 2–3 and decreasing from pH 5 to 10 (Fig. 1D). Proteinase treatment of *S. salivarius* DPC6487 CFS resulted in the loss of antimicrobial activity (Fig. 1E) as previously reported (6), and taken together, the results indicate that the antimicrobial being produced was heat stable, proteinaceous in nature, and retained activity in acidic environments.

### Whole-genome sequencing of *S. salivarius* DPC6487 revealed a gene cluster encoding a natural nisin variant

**Fig 2:**
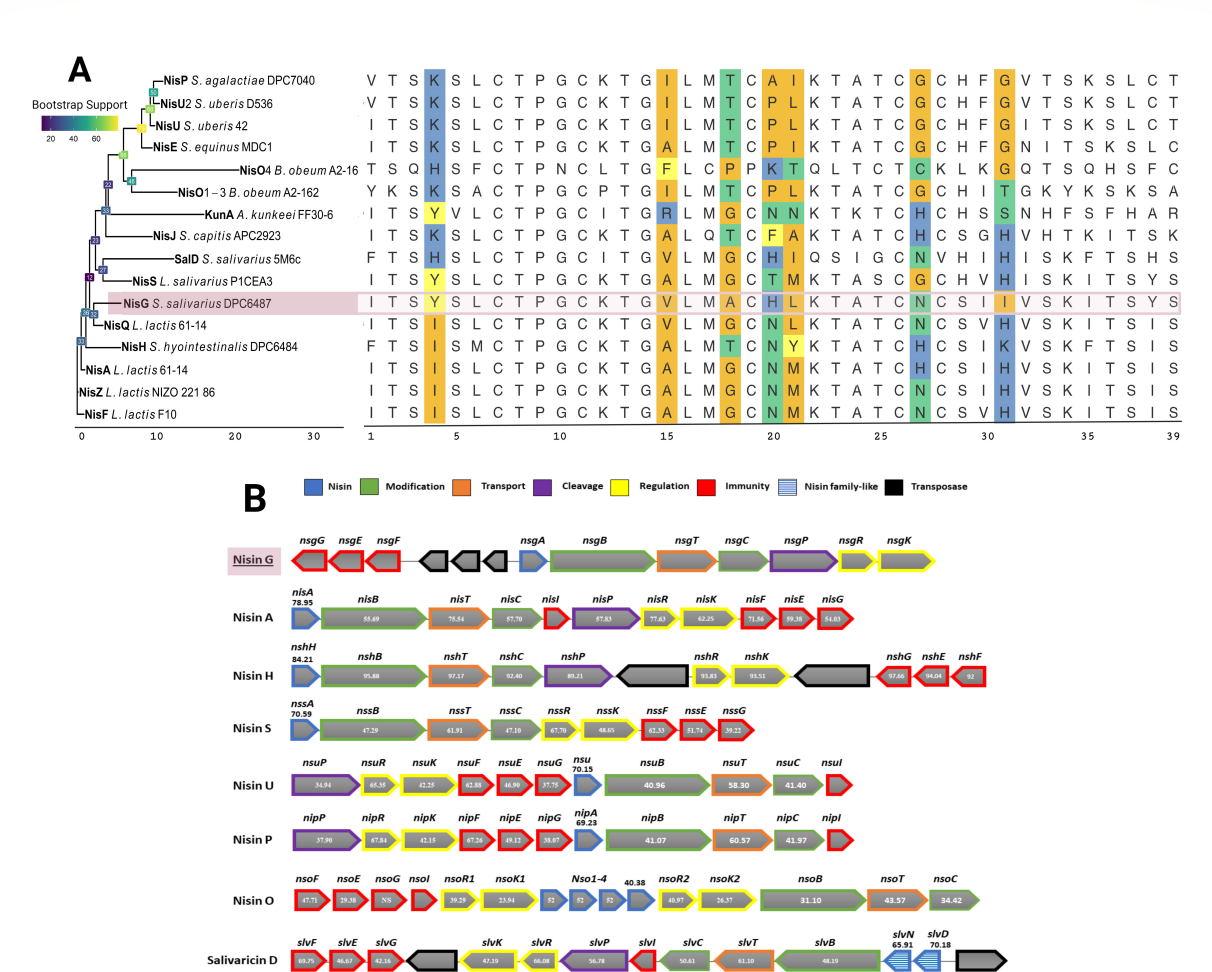
Comparison of the nisin G gene cluster found in the genome of *S. salivarius* DPC6487 with nisin A, nisin H, nisin S, nisin U, nisin P, nisin O, and salivaricin D gene clusters. (**A**) Neighbor-joining (NJ) tree and multiple sequence alignment of nisin G and other nisin variants. Branches on the NJ tree are labeled with protein and producer isolate names, with Nisin G (NisG) highlighted. Salivaricin D (SalD) produced by *S. salivarius* 5M6c and Kunkecin A (KunA) produced by *Apilactobacillus kunkeei* FF30-6 were included as they are considered “nisin like.” Internal nodes are labeled and colored by bootstrap values, based on 100 rounds of bootstrapping. Branch length (x-axis) indicates the number of amino acid substitutions per site. Nis = Nisin; Sal = Salivaricin; Kun = Kunkecin. In the multiple sequence alignment (MSA), substituted positions with respect to NisinA are highlighted (Ile4Tyr, Ala15Val, Gly18Ala, Asn20His, Met21Leu, His27Asn, and His31Ile). (**B**) Figures shown in nisin A, nisin H, and salivaricin D gene clusters represent percentage amino-acid identity to nisin G gene homologs. Where no percentage identity is indicated, no homolog was identified in the nisin G cluster. NS, no similarity. Nisin accessions (reference is provided where accession is not linked to primary source): Nisin A, ABN45880; Nisin Z, ABV64387; Nisin F, ABU45463; Nisin Q, ADB43136; Nisin H, AKB95119; Nisin J (41); Nisin U, Q2QBT0; Nisin U2, ABO32538; Nisin P (42); Nisin O (43); Kunkecin A (44), Salivaricin D, and AEX55166. Nisin E (18) and Nisin S (17).

Sequencing of *S. salivarius* DPC6487 yielded a 2,240,822 bp draft genome with an overall GC content of 39.71%. BLAST analysis of the 16S rRNA gene confirmed a 99% identity to *S. salivarius* 16S rRNA gene sequences. Average nucleotide identity (ANI) analysis against the reference genome *S. salivarius* JIM8777 resulted in an ANI value of 96.4%, which surpasses the species assignment threshold of 95% (40). ANI clustering of *S. salivarius* DPC6487 against 647 *S*. *salivarius* genomes obtained from NCBI data sets further supports its assignment as *S. salivarius* with an ANI of 95.8% and an ANI > 98.5% against 11 *S*. *salivarius* genomes. Analysis of contigs by the bacteriocin mining tool BAGEL4 indicated the presence of a potential nisin variant. Sequencing analysis confirmed that the genome of *S. salivarius* DPC6487 harbored a gene predicted to encode a natural nisin variant that was designated nisin G. Multiple sequence alignment of the putative novel nisin G peptide to other natural nisin variants revealed the following amino acid substitutions relative to nisin A: Ile4Tyr, Ala15Val, Gly18Ala, Asn20His, Met21Leu, His27Asn, and His31Ile (Fig. 2A). Nisin G is most similar to nisin Q (produced by *L. lactis* 61–14) and shares three amino acid substitutions when compared to nisin A: Ala15Val, Met21Leu, and His27Asn, while differing with respect to five amino acids: Ile4Try, Gly18Ala, Asn20His, Val30Ile, and His31Ile. Interestingly, nisin H, produced by *S. hyointestinalis*, does not share any common amino acid substitutions with nisin G when compared to nisin A. However, amino acids at positions 18, 21, and 31 are changed in both variants. Nisin G contains three unique amino acids when compared to all-natural nisin variants, i.e., Gly18Ala, Asp20His, and His31Ile (Fig. 2A).

A neighbor-joining phylogenetic tree of mature peptide sequences of natural nisin variants demonstrated broad clustering of respective streptococcal and lactococcal nisin variants, with nisin G grouping more closely with lactococcal variants. The *Blautia*-derived nisin O_1–4_, KunA (*Apilactobacillus kunkeei*), and salivaricin D are more distantly related, as shown by greater branch lengths and substitutions in the MSA. The two most recently discovered (at the time of this study) natural variants, nisin E and nisin S, are most closely related to nisin U and salivaricin D, respectively (Fig. 2A). Nisin G is predicted to be cationic at neutral pH (isoelectric point of 8.3) and water soluble.

### Genomic analysis of *S. salivarius* DPC6487 identified genes putatively responsible for the production of and immunity to nisin G

Genomic analysis identified nisin-related genes on a single-assembled contig from the draft genome of *S. salivarius* DPC6487. The nisin G encoding gene (designated as *nsgA*) and associated biosynthesis and immunity genes were identified in a gene cluster spanning a region of 14.7 kb. The putative immunity determinants displayed a similar organization to other nisin gene clusters including nisin U (15), nisin O (43), and nisin P (5) gene clusters (Fig. 2B). However, a notable feature of the nisin G genetic organization is that *nsgFEG* is transcribed in the opposite direction to *nsgA*. Streptococcal transposases were found between the nisin G encoding gene *nsgA* and *nsgF*. A region encoding transposases was also evident outside the nisin G gene cluster adjacent to the immunity determinant *nsgG* (data not shown). The presence of transposases is also common in the genetic organization with the cluster encoding the *S. hyointestinalis-*associated nisin, where a transposase is located between *nshP* and *nshR* and between *nshK* and *nshG* (16) (Fig. 2B). A transposase is also found between *slvG* and *slvK* and adjacent to *slvD* in the salivaricin D genetic organization (19). Similar to nisin H, nisin J, and nisin S, the nisin G gene cluster lacks an equivalent to the immunity gene *nisI*, and BLAST analysis against all contigs of *S. salivarius* DPC6487 genome indicated the absence of a *nisI* homolog. Therefore, the organization of nisin G gene cluster is *nsgGEFABTCPRK* (Fig. 2A). The predicted nisin G pre-peptide sequence is composed of 57 amino acids containing a leader sequence of 23 amino acids. The nisin G mature peptide displays 78.95% and 84.21% amino acid identity to nisin A and nisin H, respectively, and only 70.18% and 70.59% to salivaricin D and nisin S, respectively. Furthermore, the nisin G biosynthesis and immunity genes demonstrated the highest amino acid identity to the products of the equivalent nisin H genes, ranging from 89% to 98% identity (Fig. 2B).

### Spectrum of inhibition of *S. salivarius* DPC6487 and *L. lactis* NZ9700

The spectrum of inhibition of nisin G-producing *S. salivarius* DPC6487 and nisin A-producing *L. lactis* NZ9700 was evaluated by deferred antagonism assay (Table 1). *L. lactis* NZ9700 demonstrated bioactivity (i.e., representing the combined impact of differences in production levels and specific activity) against all *Streptococcus* species; however, of the streptococci tested, *S. salivarius* DPC6487 was only active against *Streptococcus thermophilus* strains DPC5473 and DPC5657 and *S. uberis* DPC4344. Notably, *S. salivarius* DPC6487 showed increased bioactivity against *S. agalactiae* ATCC13813 compared to the activity against other *Streptococcus* strains. In contrast to *L. lactis* NZ9700, *S. salivarius* DPC6487 showed no bioactivity against *Streptococcus mutans* strains DPC6160 and DPC6161 or *Streptococcus simulans* APC3482. *S. salivarius* DPC6487 did not demonstrate activity against the *Lactobacillus*, *Listeria*, or *Staphylococcus* strains tested here, which contrasted with the distinct antimicrobial activity observed when *L. lactis* NZ9700 was used to target these genera. As nisin G-producing *S. salivarius* DPC6487 originally showed antimicrobial activity against *F. nucleatum* DSM15643, it was hypothesized that activity would be observed against other *Fusobacterium* species and *F. nucleatum* strains. When tested, it was established that *S. salivarius* DPC6487 and *L. lactis* NZ9700 both showed antimicrobial activity against *F. nucleatum* strains DSM15643, DSM19508, and DSM19507. *Fusobacterium periodonticum* DSM19545 was also susceptible to *S. salivarius* DPC6487 and *L. lactis* NZ9700; however, no activity was observed against *Escherichia coli* K12 or *E. coli* ATCC25927. It was also established that *L. lactis* NZ9700, but not *S. salivarius* DPC6487, demonstrated bioactivity against *Clostridioides difficile* DPC6357 (Table 1).

**TABLE 1:** Indicators used in the inhibitory activity spectrum assessment of *S. salivarius* DPC6487 and *L. lactis* NZ9700 and the degree of inhibition^*a*^

	Inhibition	
Indicator	*S. salivarius* DPC6487 (nisin G producer)	*L. lactis* NZ9700 (nisin A producer)
*F. nucleatum* DSM15643	++	++
*F. nucleatum* DSM19507	++	++
*F. nucleatum subsp. vincentii* DSM19508	++	++
*F. periodonticum* DSM19545	++	++
*E. coli* K12	–	–
*E. coli* ATCC25927	–	–
*C. difficile* DPC6357	–	+
*Streptococcus mutans* DPC6160	–	+
*Streptococcus mutans* DPC6161	–	+
*Streptococcus thermophilus* DPC5472	+	+++
*Streptococcus thermophilus* DPC5657	+	+++
*Streptococcus agalactiae* ATCC13813	+++	+++
*S. uberis* DPC4344	+	+
*Streptococcus simulans* APC3482	–	+++
*Lactobacillus delbrueckii* subsp. *bulgaricus* DPC5383	+++	+++
*Limosilactobacillus fermentum* DPC3320	–	+
*Lactiplantibacillus plantarum* DPC6667	–	+++
*Staphylococcus aureus* DPC7016	–	++
Methicillin-resistant *Staphylococcus aureus* DPC5654	–	+++
*Staphylococcus epidermidis* DPC5990	–	+++
*Listeria monocytogenes* DPC3564B	–	+++
*Listeria monocytogenes* DPC3853B	–	++
*Listeria innocua* DPC3572	–	+

^*a*^ Antimicrobial activity was measured around single colonies and relative sensitivity determined as the diameter of the zone of inhibition (17). +: zones of size <2 mm, ++: zones of size 2–5 mm, and +++: zones of size >5 mm; –, no zone. DPC, Teagasc Culture collection; APC, APC Culture Collection; DMS, German Collection of Microorganisms; ATCC, American Type Culture Collection.

### Impact of the nisin G-producing *S. salivarius* DPC6487 on *F. nucleatum* in a model colonic environment

**Fig 3:**
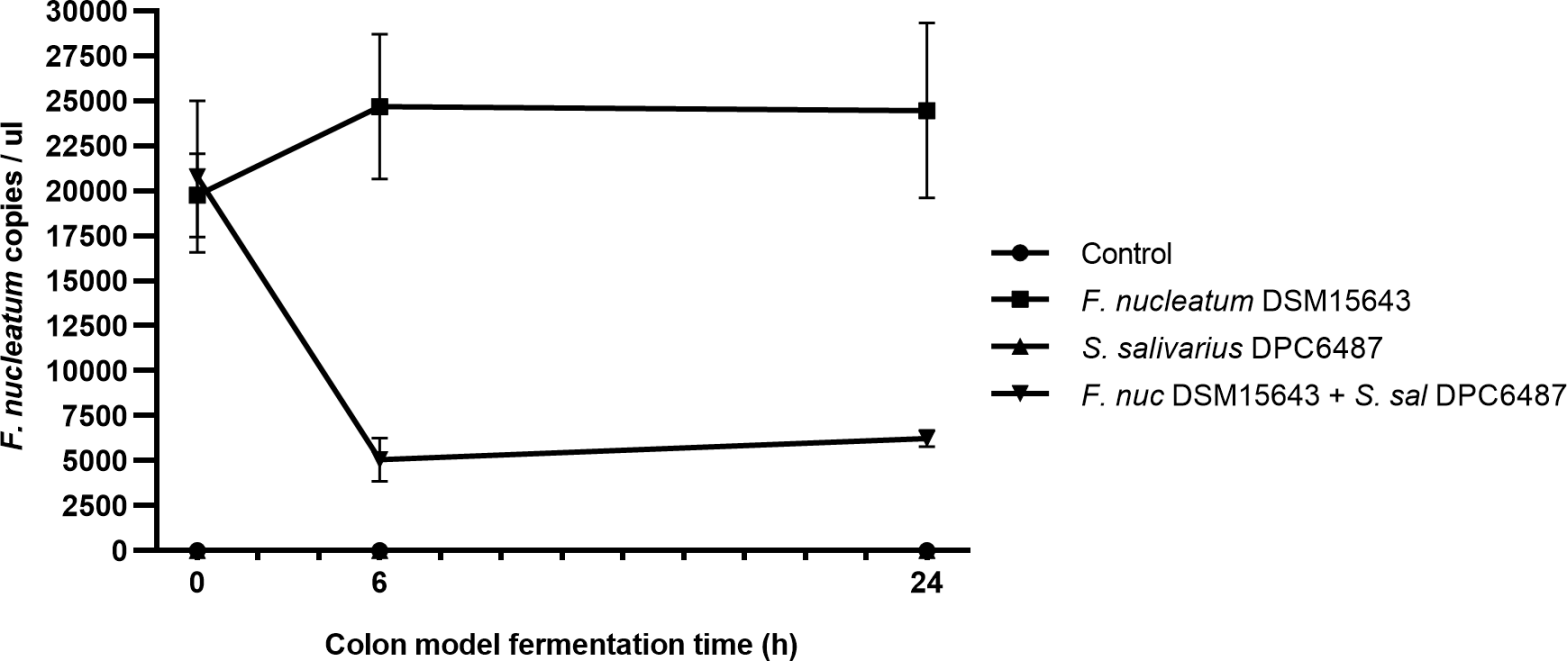
Quantification of *F. nucleatum* in colon model wells at 0, 6, and 24 hours. *F. nucleatum* numbers were significantly decreased in colon model wells that had been simultaneously inoculated with *S. salivarius* DPC6487 compared to wells inoculated with *F. nucleatum* DSM15643 alone. Mean *F. nucleatum* copy numbers and SDs for each treatment were derived from three colon model wells at each timepoint.

The impact of the nisin G-producer *S. salivarius* DPC6487 on *F. nucleatum* DSM15643 in a simulated colon environment was assessed. Quantitative PCR (qPCR) analysis revealed that at 0 hours (T0), the numbers of *F. nucleatum* were similar in samples that had been simultaneously inoculated with *S. salivarius* DPC6487 + *F. nucleatum* DSM15643 compared to those inoculated with *F. nucleatum* DSM15643 alone (19758.8 ± 2310.1 vs 20,797 ± 4214.3 Fn copies/μL, *P* = 0.7270). However, after 6 hours (T6), there was a significant drop in the numbers of *F. nucleatum* in samples that had been simultaneously inoculated with *S. salivarius* DPC6487 + *F. nucleatum* DSM15643 compared to those inoculated with *F. nucleatum* DSM15643 alone (5071.5 ± 1197.4 vs 24692.3 ± 4026.2 Fn copies/μL, *P* = 0.00126; Fig. 3). When *F. nucleatum* DSM15643 only was injected into the colon model, a 1.3-fold increase was observed after 6 hours (T6; 19758.8 ± 2310.1 to 24692.3 ± 4026.2 Fn copies/μL). However, when both *F. nucleatum* DSM15643 and *S. salivarius* DPC6487 strains were simultaneously injected into the colon model, *F. nucleatum* numbers sharply decreased (20797.8 ± 4214.3 to 5071.5 ± 1197.4 Fn copies/μL). After 24 hours (T24), *F. nucleatum* numbers remained at significantly lower levels in samples that had been simultaneously inoculated with *S. salivarius* DPC6487 + *F. nucleatum* DSM15643 compared to those inoculated with *F. nucleatum* DSM15643 alone (6237.0 ± 449.0 vs 24473.2 ± 4867.3 Fn copies/μL, *P* = 0.002958; Table S2).

### Impact of *S. salivarius* DPC6487 on compositional and functional diversity of the *ex vivo* colonic community

**Fig 4:**
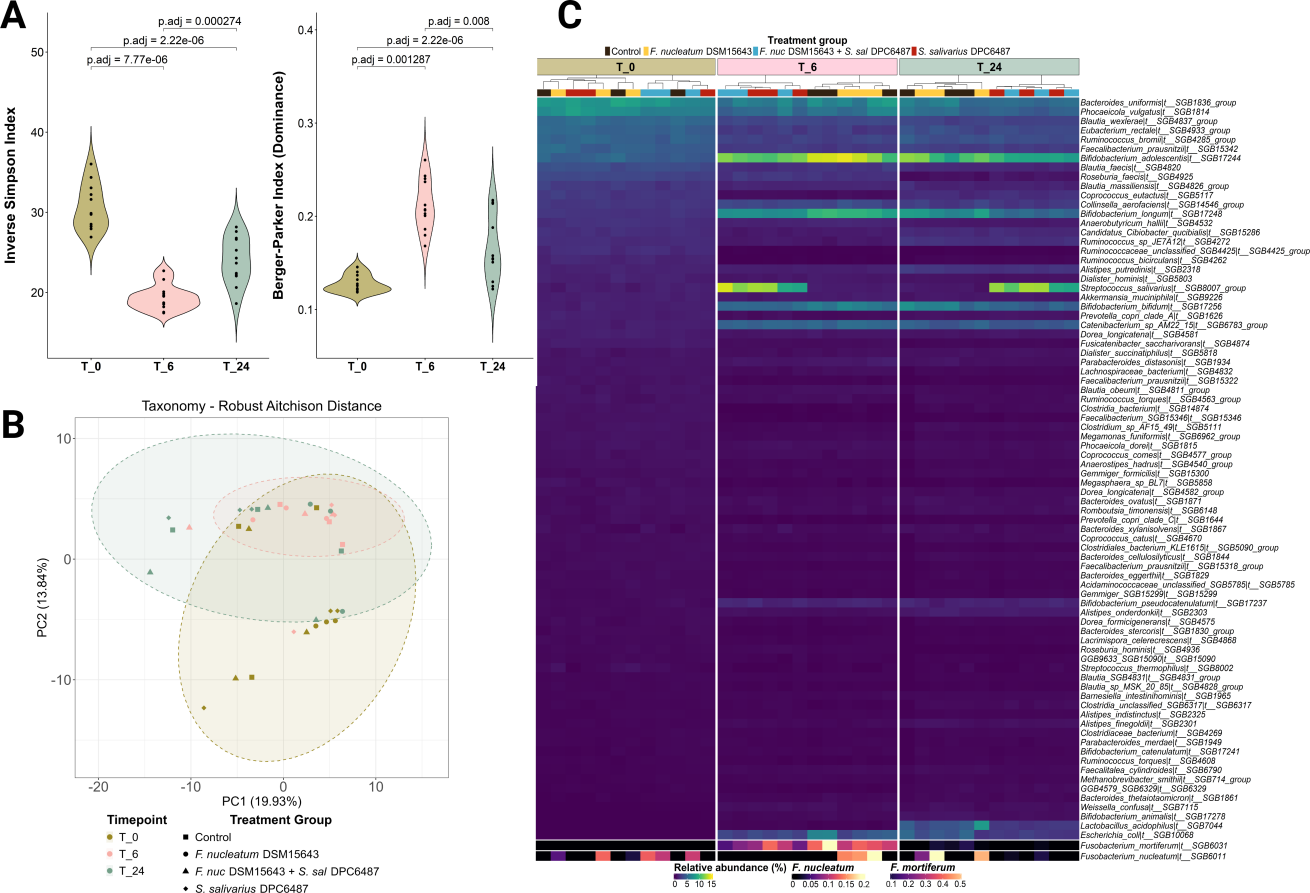
Taxonomic α-diversity (**A**), β-diversity (**B**), and relative abundances (**C**) across timepoints (T_0, T_6, and T_24). (**A**) Violin plots presenting Inverse Simpson Index values (α-diversity) and Berger-Parker Index values (dominance) at each timepoint. *P* values refer to Wilcoxon rank-sum tests and are adjusted for multiple testing. (**B**) PCoA of robust Aitchison distance matrices illustrates beta diversity of relative abundances of microbial taxa between groups. (**C**) Heatmap presents relative abundances of species within each sample (column), faceted by sampled timepoint. The main heatmap shows species present at a cumulative abundance >5% across all samples, while the bottom two rows show abundances for *Fusobacterium mortiferum* and *F. nucleatum*, which were present at relative abundances <1%.

Diversity metrics varied over the course of the experiment, with most significant separations occurring between timepoints. Across all treatments, alpha diversity declined significantly after 6 hours, with a partial recovery by 24 hours, with the inverse pattern seen for dominance (Fig. 4A). Principal coordinate analysis (PCoA) of robust Aitchison distance matrices found significant variation in beta diversity between timepoints (adonis2 permutational multivariate analysis of variance [PERMANOVA] *P* < 0.001; Fig. 4B).

Mean relative abundances of *F. nucleatum* in wells inoculated with *F. nucleatum* DSM15643 alone increased from 0.07% to 0.20% after 6 hours, reaching 0.21% at 24 hours (Fig. 4C; Table 2A). In wells co-inoculated with *S. salivarius* DPC6487, relative abundance of *F. nucleatum* decreased from 0.11% to 0.02% after 6 hours, subsequently increasing to 0.02% at T_24 (Table 2A; Fig. 4C).

**TABLE 2:** Mean relative abundances of *F. nucleatum* at each sampled timepoint, and taxa identified as dominant (*S. salivarius* and *Bifidobacterium longum*) after 24 hours for each treatment group^*a*^

Treatment	Timepoint	Mean relative abundance (%, *n* = 3)	SD
Relative abundance of *F. nucleatum* across all timepoints			
Control	T_0	0	0
	T_6	0	0
	T_24	0	0
*F. nucleatum* DSM15643 + *S. salivarius* DPC6487	T_0	0.11	0.013
	T_6	0	0
	T_24	0.02	0.013
*F. nucleatum* DSM15643	T_0	0.07	0.052
	T_6	0.2	0.088
	T_24	0.21	0.166
*S. salivarius* DPC6487	T_0	0	0
	T_6	0	0
	T_24	0	0
Relative abundance of *S. salivarius* at T_24			
Control	T_24	0.96	0.13
*F. nucleatum* DSM15643	T_24	0.79	0.18
*F. nucleatum* DSM15643 + *S. salivarius* DPC6487	T_24	10.95	2.03
*S. salivarius* DPC6487	T_24	11.62	2.12
Relative abundance of *B. longum* at T_24			
Control	T_24	7.3	0.95
*F. nucleatum* DSM15643	T_24	8.04	0.71
*F. nucleatum* DSM15643 + *S. salivarius* DPC6487	T_24	4.77	0.11
*S. salivarius* DPC6487	T_24	5.45	0.2

^*a*^ Abundances were estimated using MetaPhlAn4 taxonomic profiles after compositional transformation and filtering to species level assignments. Mean and SD are presented for three replicates.

**Fig 5:**
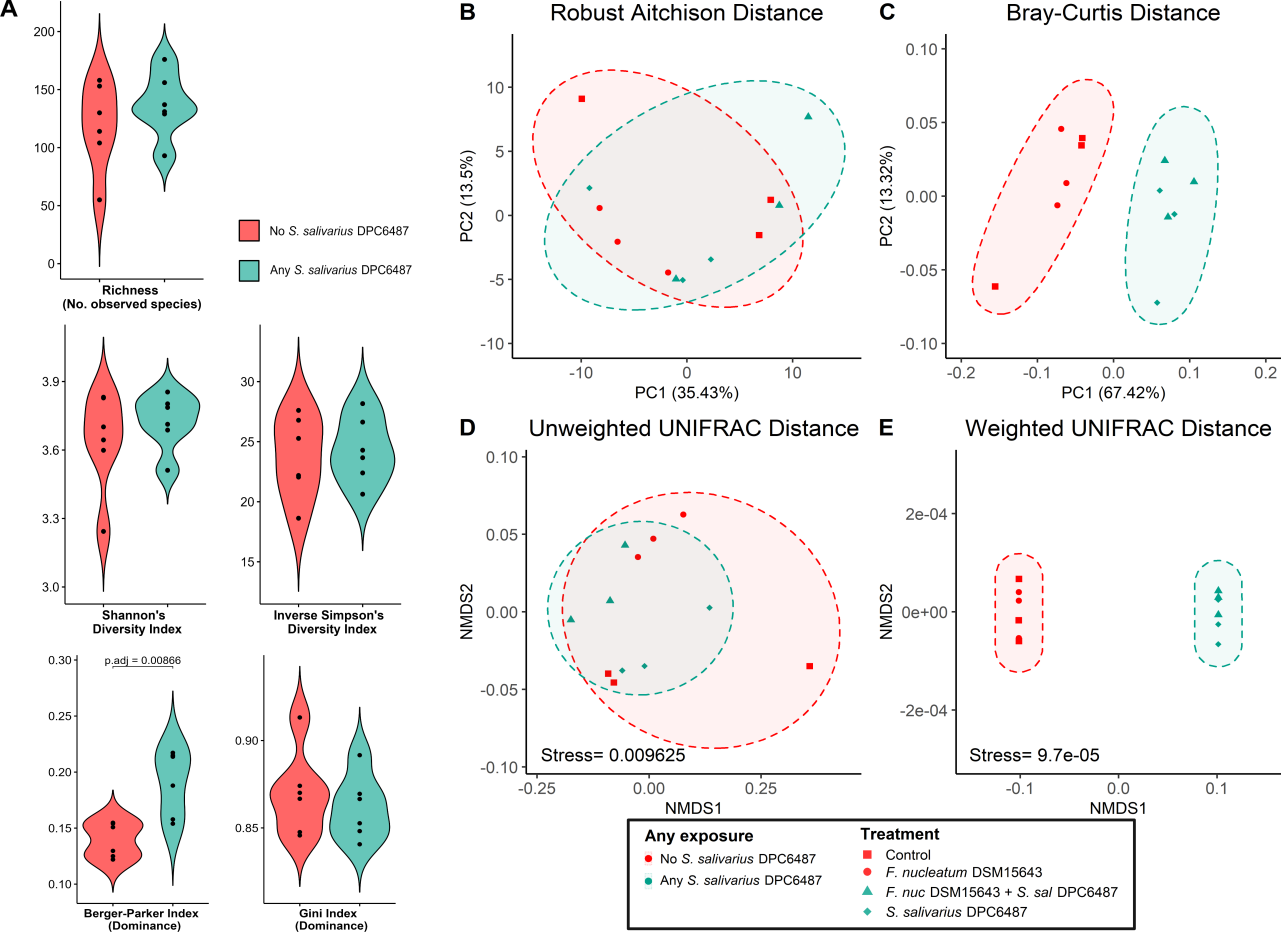
Comparison of microbiome diversity after 24 hours of treatment with *S. salivarius* DPC6487. (**A**) Violin plots comparing α-diversity metrics between wells with any *S. salivarius* DPC6487 exposure (wells inoculated with *S. salivarius* DPC6487 alone or with a combination of *S. salivarius* DPC6487 and *F. nucleatum* DSM15643) and no *S. salivarius* DPC6487 exposure (Control or inoculated with *F. nucleatum* DSM15643 alone). (**B and C**) PCoA of microbial taxonomic profiles between *S. salivarius* DPC6487-treated and untreated wells using robust Aitchison and Bray-Curtis dissimilarity matrices. (**D and E**) Non-metric multidimensional scaling (NMDS) ordination plots between *S. salivarius* DPC6487-treated and untreated wells using (**C**) unweighted UNIFRAC and (**D**) weighted UNIFRAC distances.

Next, we assessed the ecological impact of *S. salivarius* DPC6487 in the modeled colonic microbiome after 24 hours. We observed no significant differences in alpha diversity between wells with any or no inoculation with *S. salivarius* DPC6487 (Fig. 5A; Table S3; richness *P* = 0.229, *F* = 1.45; Shannon *P* = 0.681, *F* = 0.17; Inverse Simpson *P* = 0.658, *F* = 0.120). Similarly, beta diversity analyses showed significant separation in microbiome composition between treatment groups using both Bray-Curtis dissimilarity and weighted UNIFRAC distance (adonis2 PERMANOVA *P* = 0.0019, *P* = 0.0023) but not robust Aitchison or unweighted UNIFRAC distances (adonis2 PERMANOVA *P* = 0.5082, *P* = 0.5222; Fig. 5B through E).

**Fig 6:**
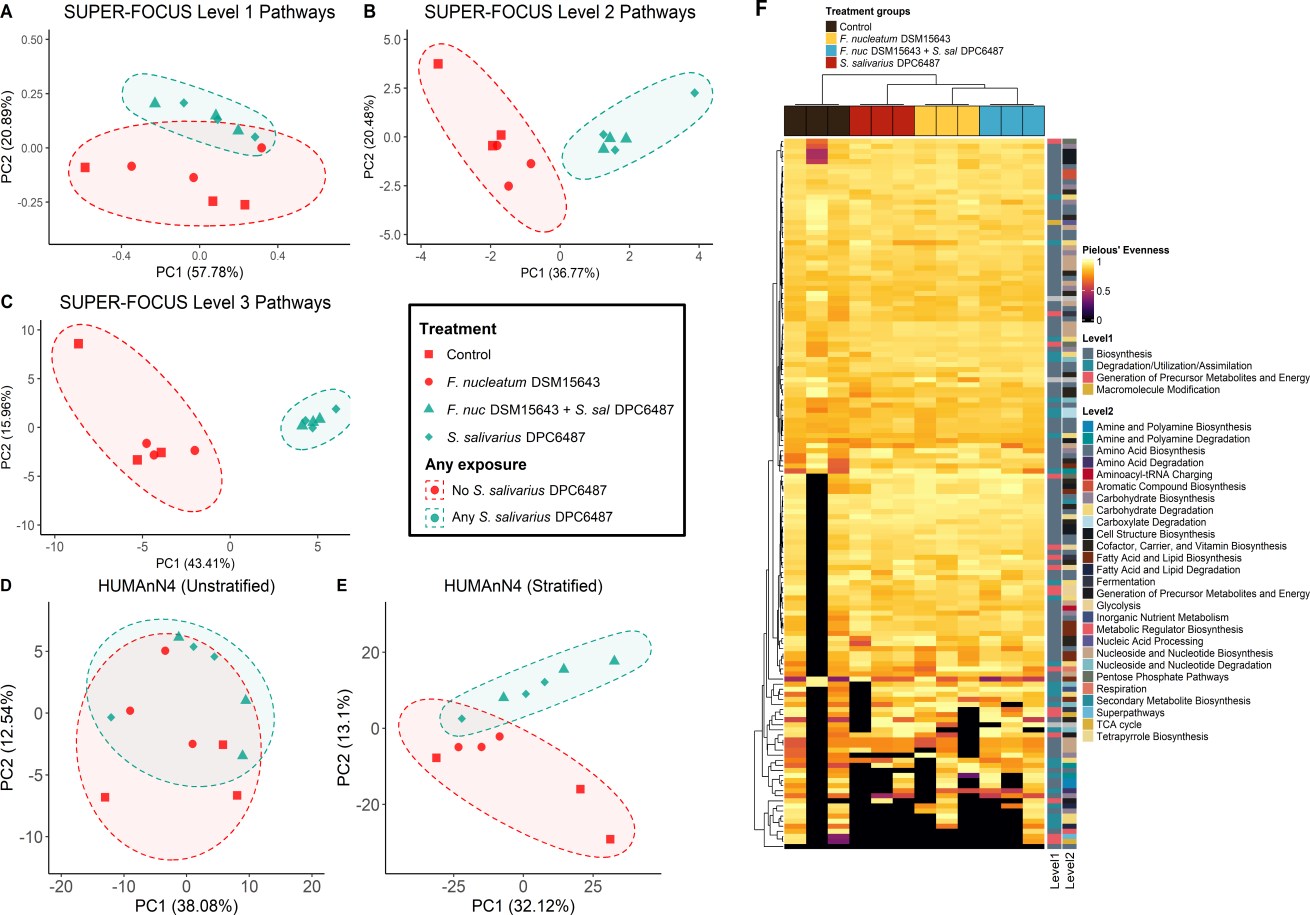
Functional profiling of samples after 24 hours. PCoA of functional profiles between *S. salivarius* DPC6487-treated and untreated wells using robust Aitchison distance matrices for (**A**) SUPER-FOCUS level 1 pathways, (**B**) SUPER-FOCUS level 2 pathways, (**C**) SUPER-FOCUS level 3 pathways, (**D**) unstratified HUMAnN4 profiles, and (**E**) stratified HUMANn4 profiles. (**F**) Contributional alpha diversity computed from stratified HUMANn4 profiles using Pielou’s evenness index, filtered to display the top 99 pathways without NA values, and samples clustered using Euclidean distances.

*S. salivarius* was dominant in *S. salivarius* DPC6487-treated wells, reaching mean relative abundances of 10.95% (*S. salivarius* DPC6487 + *F. nucleatum* DSM15643) and 11.62% (*S. salivarius* DPC6487), respectively (Table 2B). *Bifidobacterium longum* dominated in untreated wells (Control or *F. nucleatum* DSM15643), at mean abundances of 7.30% and 8.03%, respectively (*n* = 3; Table 2B). Despite being displaced as the most abundant taxon, *B. longum* persisted in *S. salivarius* DPC6487-treated wells at mean abundances of 4.77% (*S. salivarius* DPC6487 + *F. nucleatum* DSM15643) and 5.45% (*S. salivarius* DPC6487; Fig. 5C; Table 2B). Multivariate analysis found that *S. salivarius* DPC6487 treatment was significantly negatively associated with *B. longum* abundance (Coef −0.581, *q*-value = 0.002), *Bifidobacterium animalis* (Coef −0.568, *q*-value = 0.009), and *Lactobacillus_acidophilus* (Coef −0.89439, *q*-value = 0.048; Table S4). Functional analysis found no significant separation between *S. salivarius* DPC6487-treated and untreated wells after 24 hours for HUMAnN4 profiles. While separation was evident at all levels of SUPER-FOCUS-mapped pathways (Fig. 6A through E), 198 of 207 statistically significant associations with *S. salivarius* DPC6487-treatment related to *Streptococcus-*specific pathways (Table S4).

No clustering by treatment group was evident for contributional alpha diversity (Pielou’s evenness index on HUMAnN4 stratified functional pathway abundance profiles; Fig. 6F).

## DISCUSSION

The use of bacteriocin-producing probiotics to target disease-associated taxa as an alternative to antibiotics is gaining ever more interest (45) with specific species such as *S. salivarius* representing promising candidates for probiotic development through targeted inhibition of pathogenic bacteria (29). Previous work from our lab demonstrated that a salivaricin producer, *S. salivarius* DPC6993, showed anti-*F. nucleatum* activity in an *ex vivo* model of the human colon highlighting the potential use of other bacteriocin producing-*S. salivarius* to target *F. nucleatum* (39). However, subsequent genomic analysis of this strain identified the presence of potential antimicrobial resistance genes (AMRs; locus tag MRR59_RS06615 and MRR59_RS06775) (39) and revealed the need to screen for strains that did not harbor potential transferable AMRs.

In this study, a novel nisin variant, designated nisin G, produced by a gut isolate of *S. salivarius*, was identified. The producing strain, DPC6487, was shown to have antimicrobial activity *in vitro* against the emerging human pathogen *F. nucleatum*. Colony mass spectrometry of *S. salivarius* DPC6487 revealed a mass of 3,405 Da, which indicated the potential secretion of a bacteriocin. Additionally, heat, pH, and proteinase sensitivity assays with *S. salivarius* DPC6487 CFS indicated that the antimicrobial being produced was likely proteinaceous. Indeed, many bacteriocins, such as lantibiotics, are heat-stable and remain active in highly acid environments (46, 47).

Genome sequencing of *S. salivarius* DPC6487 revealed an operon with a gene encoding a novel nisin variant. This nisin variant contains seven amino acid substitutions compared to nisin A (48) and has three amino acid substitutions that are distinct from all other reported natural nisin variants; an alanine at position 18, a histidine at position 20, and an isoleucine at position 31. While streptococcal-derived nisin variants are common, for example, *S. agalactiae*, *S. uberis*, *S. hyointestinalis*, and *S. equinus* produce the variants nisin *P* (5), nisin U (15), nisin H (16), and nisin E (18), respectively, this is the first nisin reported from the species *S. salivarius*. Interestingly, a nisin-like peptide, salivaricin D, has also been reported in *S. salivarius* 5M6c (19). However, salivaricin D differs greatly from nisin A, i.e., by 14 amino acids, contains a unique region of six adjacent amino acids (HIQSIG) and comprises only four ring structures, lacking the region referred to as ring E that is characteristic of nisin molecules (19). The physiochemical properties of nisin G are predicted to be similar to that of nisin A (49, 50). Subsequent genomic analysis identified the presence of genes, *blpU* and *blpK*, as potentially encoding bacteriocins. However, masses corresponding to these bacteriocins were not detected. Indeed, natural production of these *blp* bacteriocins has been reported in a limited number of strains (51).

CRC-associated *F. nucleatum* represents a potential therapeutic target, and eradicating or suppressing the growth of this pathogen within the human gut microbiome may ultimately contribute to reducing or removing the overall risk of disease development (13). Both nisin producers *S. salivarius* DPC6487 and *L. lactis* NZ9700 displayed distinct bioactivity against *F. nucleatum* DSM15643. A limited number of studies have evaluated the *in vitro* antimicrobial activity of nisin against oral pathogens, which included *F. nucleatum* (52, 53). Overall, *S. salivarius* DPC6487 showed a narrower spectrum of activity compared to *L. lactis* NZ9700, using the overlay method, with activity against just *Fusobacterium* spp. and other streptococci. Furthermore, *S. salivarius* DPC6487 showed no activity against *C. difficile* or *E. coli* strains tested in this study, indicating a potentially narrow spectrum of activity. Narrow-spectrum antimicrobials are of particular interest as alternatives to antibiotics as they leave the surrounding microbiota unharmed (54). Both *S. salivarius* DPC6487 and *L. lactis* NZ9700 inhibited two additional *F. nucleatum* strains DSM19508 and DSM19509 and an *F. periodonticum* strain DSM19545, further supporting the anti-*Fusobacterium* potential of nisin and nisin producers. In addition, it was noted that *S. agalactiae* ATCC13813 was most susceptible of all streptococci tested to *S. salivarius* DPC6487. As invasive infections caused by *S. agalactiae* have been reported in pregnant women, new-borns, and adults with immunosuppressive diseases (55), this finding suggests a possible application for *S. salivarius* DPC6487 to reduce the risk of *S. agalactiae*-associated illnesses. As expected, the nisin A-producing *L. lactis* NZ9700 showed a broad spectrum of activity with activity against *Fusobacterium*, *Clostridioides*, *Streptococcus*, *Lactobacillus*, *Staphylococcus*, and *Listeria,* while no activity was observed against Gram-negative *E. coli* strains, as previously reported for nisin variants (5, 16). The narrower spectrum of activity observed for *S. salivarius* DPC6487 may be a consequence of the unique amino acid substitutions present in nisin G; however, further analysis is required to support this. Indeed, site-directed mutagenesis of the hinge region of nisin A resulting in either histidine at position 20 or leucine at position 21 (which are observed naturally in nisin G) reduced the bioactivity of the peptide against Gram-positive pathogens (56). Therefore, histidine in position 20 together with leucine in position 21 of the nisin G molecule may be contributing to the decreased potency observed compared to nisin A. Further analysis is required to fully understand the effect of these amino acid substitutions on the activity of nisin G.

These *in vitro* results demonstrated the potential to use *S. salivarius* DPC6487 to target *F. nucleatum* in the human gut. As *S. salivarius* DPC6487 was isolated from the human gut, it is therefore more likely to be active against *F. nucleatum* in its complex natural environment. We simulated the distal colon environment using a fecal standard (57) and used both qPCR and shotgun DNA sequencing to quantify the impact of *S. salivarius* DPC6487 on *F. nucleatum* and the surrounding microbiota. qPCR analysis revealed that the nisin-G producing *S. salivarius* strain was active in a model colonic environment, significantly reducing the growth of *F. nucleatum*, supported by subsequent metagenomic sequencing wherein abundances of the *F. nucleatum* were reduced in colon model wells inoculated with *S. salivarius* DPC6487 and *F. nucleatum* DSM15643 compared to wells inoculated with *F. nucleatum* DSM15643 alone. While these results provide strong evidence for the antimicrobial activity of nisin G-producing *S. salivarius* DPC6487 against *F. nucleatum*, the antagonistic effect may also be due to other factors such as niche competition.

Although probiotics that produce bacteriocins with antimicrobial activity against specific microbial pathogens are a potential alternative to antibiotics, narrow-spectrum antimicrobials are especially desirable, as broad-spectrum antimicrobials may negatively impact the gut microbiome (58). We investigated the ecological impact of the nisin G-producing *S. salivarius* DPC6487 at probiotic dosage in the colon model. Overall, the most significant separations occurred between sampled timepoints, which is likely due to limitations of the *in vitro* distal colon model environment that have been reported in similar studies (59). We found no significant differences in α-diversity between wells to which *S. salivarius* DPC6487 had been treated and those in which it had not; furthermore, significant separation in terms of taxonomic β- diversity was only observed for ordinations using distance metrics that emphasize abundance (Bray-Curtis, weighted UNIFRAC). Multivariate analysis of the taxonomic and functional profiles highlighted that differences in functional diversity were largely attributable to pathways mapping to the genus *Streptococcus.* Contributional alpha diversity, which assesses the variety and evenness of taxa contributing to a pathway in a sample, did not show evident clustering by treatment with *S. salivarius* DPC6487. Taken together, the separation and apparent dominance of *S. salivarius* may relate to the probiotic dosage applied in this model (10^9^ CFU/mL) but did not significantly impact the overall taxonomic and functional diversity of the modeled distal colon community. At an individual species level, we did find negative associations between *S. salivarius* DPC6487 and certain species of bifidobacteria, which may relate to indirect competition or the high inoculum of *S. salivarius* inflating the overall microbial load and impacting relative abundances. The *in vitro* activity against *F. periodonticum* may indicate a broader activity of nisin G against fusobacterial species.

In conclusion, numerous studies have used nisin as a means of targeting disease-associated taxa (22). A long safety record, limited risk for resistance development, activity against antibiotic resistance microbes, bioengineering potential, and cost-effective scalability further support nisin’s therapeutic use for the treatment of bacterial infections (60, 61). As such, nisin-producing bacteria represent potential candidates for the development of antimicrobial-producing probiotics, which may be utilized to target pathogenic bacteria, and consequently, lower the overall risk of disease development. The nisin G-producing *S. salivarius* DPC6487 is a candidate for probiotic development with potential application to target the CRC-associated pathogen *F. nucleatum. In vivo* trials and safety assessments on*S. salivarius* DPC6487 are needed to provide stronger evidence and support the potential of *S. salivarius* DPC6487 as a probiotic.

## MATERIALS AND METHODS

### Bacterial strains and cultivation media

*S. salivarius* DPC6487, previously isolated from a neonatal fecal sample, was sourced from the Teagasc culture collection (6). The strain was cultivated under anaerobic conditions at 37°C in brain heart infusion (Difco Laboratories, Detroit, MI, USA) broth and agar medium containing 1.5% (wt/vol) agar. Anaerobic conditions were simulated using anaerobic jars with Anaerocult A gas packs (Merck, Darmstadt, Germany) or a Don Whitley Anaerobic workstation (nitrogen 85%, carbon dioxide 5%, and hydrogen 10%). A full list of bacteria and their culture conditions used in this study are presented in Table S1.

### Colony mass spectrometry of *S. salivarius* DPC6487

Fully grown colonies of *S. salivarius* DPC6487 were mixed with 50 µL 2-propanol 0.1% trifluoroacetic acid (TFA), vortexed three times, and centrifuged at 21,300 g for 30 seconds. MALDI TOF mass spectrometry was performed on the cell-free supernatant using an Axima TOF^2^ MALDI-TOF mass spectrometer (Shimadzu Biotech, Manchester, UK). A 0.5 µL aliquot of matrix solution (α-cyano 4-hydroxy cinnamic acid, 10 mg/mL in acetonitrile—0.1% [vol/vol] TFA) was deposited onto the target and left for 5 seconds before being removed. The residual solution was allowed to dry, and 0.5 µL sample solution was deposited onto the pre-coated sample spot. A 0.5 µL aliquot of matrix solution was added to the deposited sample and allowed to dry. The sample was subsequently analyzed in positive-ion linear mode.

### Antimicrobial activity assays

Antimicrobial activity of *S. salivarius* DPC6487 and *L. lactis* NZ9700 against *F. nucleatum* DSM15643 and a range of indicator strains was determined by a deferred antagonism assay (62). In brief, a fully cultured nisin-producer streak plate was overlayed with 0.75% agar seeded with the indicator microorganism and incubated according to the growth condition of the indicator microorganism (Table S1). Inhibitory activity of *S. salivarius* DPC6487 CFS against *L. bulgaricus* DPC5383 was determined by well diffusion assay (WDA) as previously described (41). Briefly, 50 µL of CFS prepared from 10 mL of an overnight culture of *S. salivarius* DPC6487 was added to wells of De Man–Rogosa–Sharpe (MRS) agar plates seeded with *L. bulgaricus* DPC5383 and incubated at 37°C under anaerobic conditions for 18–20 hours. Antimicrobial activity was determined by the presence of a zone of inhibition.

### Heat, pH, and protease sensitivity assays

The stability of the antimicrobial secreted by *S. salivarius* DPC6487 to a variety of physio-chemical factors was examined. Initially, CFS was subjected to temperatures of 37°C, 60°C, 70°C, 80°C, 90°C, 100°C, and 121°C for 10 minutes, and the heat-treated CFS was evaluated for its antimicrobial activity against *L. bulgaricus* DPC5383 by WDA, as previously described. A pH stability test was performed by altering the pH of *S. salivarius* DPC6487 CFS to 2.0, 3.0, 5.0, 8.0, and 10.0 by the addition of 1 M HCL or 1 M NaOH. Antimicrobial activity of the pH-adjusted CFS against *L. bulgaricus* DPC5383 was evaluated by WDA. A proteinase sensitivity assay was performed by incubating the *S. salivarius* DPC6487 CFS with 20 mg/mL proteinase K (Sigma) at 37°C for 1 hour. CFS mixed with an equal volume of molecular biology-grade water was included as a control. The antimicrobial activity of these CFSs was determined against *L. bulgaricus* DPC5383 by WDA.

### *S. salivarius* DPC6487 whole-genome sequencing and analysis

Genomic DNA (gDNA) was extracted from *S. salivarius* DPC6487 culture cell pellets using a GenElute Bacterial Genomic DNA Kit (Sigma-Aldrich; Co. Wicklow, Ireland). The purity and concentration of genomic DNA were confirmed using the NanoDrop 1000 (ThermoFisher Scientific, Dublin, Ireland) and Qubit 2.0 Fluorometer (ThermoFisher Scientific, Dublin, Ireland) according to the respective protocols. DNA was prepared according to the Nextera XT DNA library preparation guide from Illumina and sequenced on an Illumina MiSeq (Teagasc, Moorepark Sequencing Facility). Quality trimming of the resulting raw FastQ files was performed using TrimGalore (v.0.6.0; URL: https://www.bioinformatics.babraham.ac.uk/projects/trim_galore/), a wrapper script for cutadapt (v. 2.6) (63), and FastQC (v. 0.11.8) (64) with a *q*-score cut-off of 30 and minimum length after trimming of 105 bp. Error correction and assembly into contigs were performed using SPAdes (v. 3.14) (65) in “isolate” run mode. The assembled contigs of the draft genome were annotated using Prokka (v. 1.14) (66) with RNAmmer (67) for rRNA prediction. BAGEL4 software, an automated bacteriocin mining tool, was used to detect the presence of putative bacteriocin operons (68). Manual analysis of the contigs was then subsequently performed using the ARTEMIS genome browser (69). The putative bacteriocin gene clusters were manually annotated following sequence similarity analyses using the BLASTp algorithm and the non-redundant database provided by the NCBI (70) (http://blast.ncbi.nlm.nih.gov).

Phylogenetic relatedness between nisin variants was inferred by aligning amino acid sequences using msaClustalW in the msa R package (v 1.30.1; BLOSUM matrix, gap opening penalty = 10, gap extension penalty = 0.2) (71). Pairwise distances were computed and neighbor-joining trees using the *dist.aa* and *njs* functions in ape (v. 5.7–1) with 100 rounds of bootstrapping. The tree and multiple sequence alignment were visualized using ggtree (v 3.6.2) and ggmsa (v.1.3.4) in R.

### Preparation of a fecal standard

The fecal slurry was prepared using a previous methodology (39, 57). Briefly, fecal samples were provided by six healthy donors with no antibiotic treatment in the previous 6 months. Participants gave written consent, as part of the study approved by the Clinical Research Ethics Committee of the Cork Teaching Hospitals, UCC, (ECM 4 [x] 22 February 2022). A total mixture of 200 g from the samples was processed in an anaerobic chamber (5% CO_2_, 10% H_2_, and 85% N_2_), after being kept at 4°C for 1–2 hours, mixing them with 200 mL saline buffer containing 0.05% (wt/vol) cysteine hydrochloride in a Circulator 400 stomacher bag (Seward, UK), previous to being homogenized, centrifuged (4,000 × *g*, 25 min), and resuspended in phosphate buffered saline. Sterile glycerol was added to a final concentration of 25%, aliquoted, and stored at −80°C.

### MicroMatrix fermentation

A fecal slurry aliquot was defrosted before use and prepared at 10% concentration in fecal fermentation medium (39, 57). Four treatments, conducted in triplicates, were applied to designated wells to a final volume of 6 mL in fermentation medium. The treatments were as follows: one inoculating *F. nucleatum* DSM154 adjusted to a concentration of approximately 10^4^ CFU/µL; one inoculating *S. salivarius* DPC6487 adjusted to a concentration of 10^9^ CFU/mL, as recommended probiotic dose (72); one combining both adjusted concentration of *F. nucleatum* DSM154 and *S. salivarius* DPC6487. Control wells containing just the fecal slurry in fermentation medium were also included. At time 0 (T0), a 1 mL aliquot was removed from each well in the anaerobic sample, reducing the final fermentation volume to 5 mL per well. Fermentations were conducted for 24  hours in a MicroMatrix fermenter (Applikon Biotechnology) with the following parameters: nitrogen gas (40%), CO_2_ gas, Orbiter (300 rpm), NaOH, pH (6.8), temperature (37°C), and DO (0%). Aliquots of 1 mL were collected from each well at 6 and 24 hours (T6 and T24) and kept at −80°C for further analysis.

### DNA extraction

gDNA was extracted from 1 mL aliquots using the QIAmp PowerFecal ProDNA kit (Qiagen, Crawley, UK). Briefly, the centrifuged samples (4,000 rpm, 10 min) were resuspended in lysis buffer, and extractions were performed according to the manufacturer’s instructions. DNA was quantified using the Qubit 2.0 Fluorometer (Life Technologies, USA), and the purity was checked using the NanoDrop 1000 (ThermoFisher Scientific, Ireland).

### Quantitative PCR

The abundance of *F. nucleatum* was determined by qPCR, using the Roche LightCycler 480 II platform, using primers amplifying the *nusG* gene (forward: 5′-CAACCATTACTTTAACTCTACCATGTTCA-3′ and reverse: 5’- GTTGACTTTACAGAAGGAGATTATGTAAAAATC-3′) (30). A standard curve was generated using 3 × 10^5^ to 3 × 10^1^ copies of the *nusG*/µL with an efficiency of 94% and an R-squared value of >0.99, using previously extracted *F. nucleatum* DSM15643 gDNA. Samples were run using KAPA Lightcycler 480 mix (KAPA Biosystems Ltd., UK) following the manufacturer’s instructions. The cycling conditions were a preincubation period of 5 min at 95°C, an amplification period of 40 cycles of 94°C for 30 seconds, 55°C for 30 seconds, and 72°C for 1 minute, and a final stage of 95°C for 5 seconds and 47°C for 1 minute. All samples were run in triplicate. The metagenomics samples were quantified against the standard curve, obtaining the number of *nusG* copies.

### Shotgun sequencing and bioinformatic analysis of sequencing data

DNA was prepared according to the Nextera XT DNA library preparation guide from Illumina and sequenced using 2 × 150 bp paired-end kit on the Illumina NextSeq Platform (Teagasc, Moorepark Sequencing Facility), with base-calling using bcl-convert (v3.8.4). Raw sequencing reads were processed through Trim Galore (v.0.6.1), with adapter and quality trimming performed using Cutadapt (v.2.6) and Fastqc (v.0.11.8), respectively, using default parameters and a Phred score cut-off of 20. Next, human host reads were filtered out by alignment to the human genome (*Homo sapiens*, hg38) using Bowtie 2 (v2.3.4). Seqkit (v.1.4) was used to verify microbial read counts after trimming and host removal. Quality trimming and host removal resulted in a mean of 3,333,975 high-quality microbial sequencing reads per sample (min 850,680 and max 8,453,750), which yielded taxonomic profiles with a median 121 observed species per sample.

Taxonomic classification was performed using MetaPhlAn4 (v.4.0, database vJan21_CHOCOPhlAnSGB_202103). Functional profiling was performed on interleaved files (bbmap.v.38.22) using HUMAnN.v.3.6, using the taxonomic profiles from MetaPhlAn4 output with Bowtie2 (v.2.4) and DIAMOND (v.2.0.15). Both bacterial and archaeal reads were considered, with bacteria accounting for >99% of assigned reads in all samples. Additional functional profiling was performed using SUPER-FOCUS (v 0.34).

### Statistical analysis

#### Quantitative PCR

Significant differences between groups were established using a paired *t*-test, assuming normal distribution, and equal variances. Both sides of the distribution were considered. Significance was considered when *P* < 0.05. Calculations were performed using Excel 365 and GraphPad Prism v 9.4.1 (GraphPad Software Inc, USA).

#### Microbiome analysis

Analysis was performed in the R statistical programming environment (v 4.2.2) and visualized using ggplot2 (73) and ComplexHeatmap (74). Alpha diversity was assessed using the vegan package (v 2.6–4) (75). Dominance indices and dominant species were calculated using the microbiome package (v 1.20.0), with compositional transformation. Between-group comparisons were performed using non-parametric Kruskal-Wallis tests, with Wilcoxon signed-rank testing for significantly different results (rstatix, v0.7.2 [76]), applying Benjamini-Hochberg correction for multiple comparisons. Due to the sample size of treatment groups at each timepoint (*n* = 3), “*S. salivarius*-exposed” (wells inoculated with *S. salivarius* alone or with a combination of *S. salivarius* and *F. nucleatum*) and “No *S. salivarius* exposure” (control wells or wells inoculated with *F. nucleatum* only) were compared within each timepoint (*n* = 6).

Dissimilarity matrices of taxonomic profiles were generated for Bray-Curtis and robust Aitchison distance (vegan), as well as weighted and unweighted UNIFRAC distance (phyloseq). These were compared using PERMANOVA (implemented via vegan::adonis2), analysis of similarity (ANOSIM; vegan::anosim), and beta dispersion (vegan::betadisper). Ordination of dissimilarity matrices for Bray-Curtis and robust Aitchison distance was performed using PCoA (vegan), while UNIFRAC distances underwent ordination with non-metric multidimensional scaling (phyloseq), with the exception of weighted UNIFRAC distances at T_24 where low stress was observed (9.9e-0.5), and hence, PCoA was applied (phyloseq).

Dissimilarity matrices were generated for functional profiles using robust Aitchison distance (vegan) and assessed as described above for taxonomic profiles (PERMANOVA, ANOSIM, and beta dispersion). Contributional alpha diversity was computed on HUMAnN4 taxonomy-stratified pathway abundance, using the FuncDiv package (v 1.0.0) (77).

## Data Availability

The sequence of the nisin G gene cluster of *Streptococcus salivarius* DPC6487 has been deposited in GenBank under accession number OR885872. The sequence data generated from the *ex vivo* colon model have been deposited in the European Nucleotide Archive under accession number PRJEB74144. Scripts used for statistical analysis and data visualization are available at https://github.com/aforestsomewhere/nisinG.

## SUPPLEMENTAL MATERIAL

Supplemental material mbio.01573-24-s0001.docx Tables S1-S4.

ASM does not own the copyrights to Supplemental Material that may be linked to, or accessed through, an article. The authors have granted ASM a non-exclusive, world-wide license to publish the Supplemental Material files. Please contact the corresponding author directly for reuse.

## References

[1] Bauer MA, Kainz K, Carmona-Gutierrez D, Madeo F. 2018. Microbial wars: competition in ecological niches and within the microbiome. Microb Cell 5:215–219. doi:10.15698/mic2018.05.628 29796386 PMC5961915

[2] Hibbing ME, Fuqua C, Parsek MR, Peterson SB. 2010. Bacterial competition: surviving and thriving in the microbial jungle. Nat Rev Microbiol 8:15–25. doi:10.1038/nrmicro2259 19946288 PMC2879262

[3] Mignolet J, Fontaine L, Sass A, Nannan C, Mahillon J, Coenye T, Hols P. 2018. Circuitry rewiring directly couples competence to predation in the gut dweller Streptococcus salivarius. Cell Rep 22:1627–1638. doi:10.1016/j.celrep.2018.01.055 29444418

[4] Stein T, Borchert S, Kiesau P, Heinzmann S, Klöss S, Klein C, Helfrich M, Entian KD. 2002. Dual control of subtilin biosynthesis and immunity in Bacillus subtilis. Mol Microbiol 44:403–416. doi:10.1046/j.1365-2958.2002.02869.x 11972779

[5] Garcia-Gutierrez E, O’Connor PM, Saalbach G, Walsh CJ, Hegarty JW, Guinane CM, Mayer MJ, Narbad A, Cotter PD. 2020. First evidence of production of the lantibiotic nisin P. Sci Rep 10:3738. doi:10.1038/s41598-020-60623-0 32111904 PMC7048740

[6] O’Shea EF, Gardiner GE, O’Connor PM, Mills S, Ross RP, Hill C. 2009. Characterization of enterocin- and salivaricin-producing lactic acid bacteria from the mammalian gastrointestinal tract. FEMS Microbiol Lett 291:24–34. doi:10.1111/j.1574-6968.2008.01427.x 19076236

[7] Lakshminarayanan B, Guinane CM, O’Connor PM, Coakley M, Hill C, Stanton C, O’Toole PW, Ross RP. 2013. Isolation and characterization of bacteriocin-producing bacteria from the intestinal microbiota of elderly Irish subjects. J Appl Microbiol 114:886–898. doi:10.1111/jam.12085 23181509

[8] Santos-Júnior CD, Torres MDT, Duan Y, Rodríguez Del Río Á, Schmidt TSB, Chong H, Fullam A, Kuhn M, Zhu C, Houseman A, Somborski J, Vines A, Zhao X-M, Bork P, Huerta-Cepas J, de la Fuente-Nunez C, Coelho LP. 2024. Discovery of antimicrobial peptides in the global microbiome with machine learning. Cell 187:3761–3778. doi:10.1016/j.cell.2024.05.013 38843834 PMC11666328

[9] Gallardo-Becerra L, Cervantes-Echeverría M, Cornejo-Granados F, Vazquez-Morado LE, Ochoa-Leyva A. 2023. Microbiota perspectives in searching antimicrobial peptides (AMPs) produced by the microbiota. Microb Ecol 87:8. doi:10.1007/s00248-023-02313-8 38036921 PMC10689560

[10] Reuben RC, Torres C. 2024. Bacteriocins: potentials and prospects in health and agrifood systems. Arch Microbiol 206:233. doi:10.1007/s00203-024-03948-y 38662051 PMC11045635

[11] Arnison PG, Bibb MJ, Bierbaum G, Bowers AA, Bugni TS, Bulaj G, Camarero JA, Campopiano DJ, Challis GL, Clardy J, et al.. 2013. Ribosomally synthesized and post-translationally modified peptide natural products: overview and recommendations for a universal nomenclature. Nat Prod Rep 30:108–160. doi:10.1039/c2np20085f 23165928 PMC3954855

[12] Chikindas ML, Weeks R, Drider D, Chistyakov VA, Dicks LM. 2018. Functions and emerging applications of bacteriocins modified title: functions and emerging applications of bacteriocins. Curr Opin Biotechnol 49:23–28. doi:10.1016/j.copbio.2017.07.011 28787641 PMC5799035

[13] Lawrence GW, Begley M, Cotter PD, Guinane CM. 2020. Potential use of biotherapeutic bacteria to target colorectal cancer-associated taxa. Int J Mol Sci 21:924. doi:10.3390/ijms21030924 32019270 PMC7037558

[14] Nes IF, Diep DB, Holo H. 2007. Streptococcus enterococcus bacteriocin diversity in Streptococcus and Enterococcus. J Bacteriol 189:1189–1198. doi:10.1128/JB.01254-06 17098898 PMC1797368

[15] Wirawan RE, Klesse NA, Jack RW, Tagg JR. 2006. Molecular and genetic characterization of a novel nisin variant produced by Streptococcus uberis. Appl Environ Microbiol 72:1148–1156. doi:10.1128/AEM.72.2.1148-1156.2006 16461661 PMC1392965

[16] O’Connor PM, O’Shea EF, Guinane CM, O’Sullivan O, Cotter PD, Ross RP, Hill C. 2015. Nisin H is a new nisin variant produced by the gut-derived strain Streptococcus hyointestinalis DPC6484. Appl Environ Microbiol 81:3953–3960. doi:10.1128/AEM.00212-15 25841003 PMC4524162

[17] Sevillano E, Peña N, Lafuente I, Cintas LM, Muñoz-Atienza E, Hernández PE, Borrero J. 2023. Nisin S, a novel nisin variant produced by Ligilactobacillus salivarius P1CEA3. Int J Mol Sci 24:6813. doi:10.3390/ijms24076813 37047785 PMC10095417

[18] Sugrue I, Hill D, O’Connor PM, Day L, Stanton C, Hill C, Ross RP. 2023. Nisin E is a novel nisin variant produced by multiple Streptococcus equinus strains. Microorganisms 11:427. doi:10.3390/microorganisms11020427 36838392 PMC9958725

[19] Birri DJ, Brede DA, Nes IF. 2012. Salivaricin D, a novel intrinsically trypsin-resistant lantibiotic from Streptococcus salivarius 5M6c isolated from a healthy infant. Appl Environ Microbiol 78:402–410. doi:10.1128/AEM.06588-11 22101034 PMC3255740

[20] Wiedemann I, Breukink E, van Kraaij C, Kuipers OP, Bierbaum G, de Kruijff B, Sahl HG. 2001. Specific binding of nisin to the peptidoglycan precursor lipid II combines pore formation and inhibition of cell wall biosynthesis for potent antibiotic activity. J Biol Chem 276:1772–1779. doi:10.1074/jbc.M006770200 11038353

[21] Hansen JN. 1994. Nisin as a model food preservative. Crit Rev Food Sci Nutr 34:69–93. doi:10.1080/10408399409527650 8142045

[22] Shin JM, Gwak JW, Kamarajan P, Fenno JC, Rickard AH, Kapila YL. 2016. Biomedical applications of nisin. J Appl Microbiol 120:1449–1465. doi:10.1111/jam.13033 26678028 PMC4866897

[23] Wescombe PA, Upton M, Dierksen KP, Ragland NL, Sivabalan S, Wirawan RE, Inglis MA, Moore CJ, Walker GV, Chilcott CN, Jenkinson HF, Tagg JR. 2006. Production of the lantibiotic salivaricin A and its variants by oral streptococci and use of a specific induction assay to detect their presence in human saliva. Appl Environ Microbiol 72:1459–1466. doi:10.1128/AEM.72.2.1459-1466.2006 16461700 PMC1392966

[24] Burton JP, Wescombe PA, Moore CJ, Chilcott CN, Tagg JR. 2006. Safety assessment of the oral cavity probiotic Streptococcus salivarius K12. Appl Environ Microbiol 72:3050–3053. doi:10.1128/AEM.72.4.3050-3053.2006 16598017 PMC1449041

[25] Wescombe PA, Hale JDF, Heng NCK, Tagg JR. 2012. Developing oral probiotics from Streptococcus salivarius. Future Microbiol 7:1355–1371. doi:10.2217/fmb.12.113 23231486

[26] Burton JP, Cowley S, Simon RR, McKinney J, Wescombe PA, Tagg JR. 2011. Evaluation of safety and human tolerance of the oral probiotic Streptococcus salivarius K12: a randomized, placebo-controlled, double-blind study. Food Chem Toxicol 49:2356–2364. doi:10.1016/j.fct.2011.06.038 21722694

[27] Di Pierro F, Colombo M, Zanvit A, Rottoli AS. 2016. Positive clinical outcomes derived from using Streptococcus salivarius K12 to prevent streptococcal pharyngotonsillitis in children: a pilot investigation. Drug Healthc Patient Saf 8:77–81. doi:10.2147/DHPS.S117214 27920580 PMC5123729

[28] Zupancic K, Kriksic V, Kovacevic I, Kovacevic D. 2017. Influence of oral probiotic Streptococcus salivarius K12 on ear and oral cavity health in humans: systematic review. Probiotics Antimicrob Proteins 9:102–110. doi:10.1007/s12602-017-9261-2 28236205

[29] Wescombe PA, Heng NCK, Burton JP, Chilcott CN, Tagg JR. 2009. Streptococcal bacteriocins and the case for Streptococcus salivarius as model oral probiotics. Future Microbiol 4:819–835. doi:10.2217/fmb.09.61 19722837

[30] Castellarin M, Warren RL, Freeman JD, Dreolini L, Krzywinski M, Strauss J, Barnes R, Watson P, Allen-Vercoe E, Moore RA, Holt RA. 2012. Fusobacterium nucleatum infection is prevalent in human colorectal carcinoma. Genome Res 22:299–306. doi:10.1101/gr.126516.111 22009989 PMC3266037

[31] Chen W, Liu F, Ling Z, Tong X, Xiang C. 2012. Human intestinal lumen and mucosa-associated microbiota in patients with colorectal cancer. PLoS One 7:e39743. doi:10.1371/journal.pone.0039743 22761885 PMC3386193

[32] Flanagan L, Schmid J, Ebert M, Soucek P, Kunicka T, Liska V, Bruha J, Neary P, Dezeeuw N, Tommasino M, Jenab M, Prehn JHM, Hughes DJ. 2014. Fusobacterium nucleatum associates with stages of colorectal neoplasia development, colorectal cancer and disease outcome. Eur J Clin Microbiol Infect Dis 33:1381–1390. doi:10.1007/s10096-014-2081-3 24599709

[33] Flemer B, Warren RD, Barrett MP, Cisek K, Das A, Jeffery IB, Hurley E, O’Riordain M, Shanahan F, O’Toole PW. 2018. The oral microbiota in colorectal cancer is distinctive and predictive. Gut 67:1454–1463. doi:10.1136/gutjnl-2017-314814 28988196 PMC6204958

[34] Kostic AD, Chun E, Robertson L, Glickman JN, Gallini CA, Michaud M, Clancy TE, Chung DC, Lochhead P, Hold GL, El-Omar EM, Brenner D, Fuchs CS, Meyerson M, Garrett WS. 2013. Fusobacterium nucleatum potentiates intestinal tumorigenesis and modulates the tumor-immune microenvironment. Cell Host Microbe 14:207–215. doi:10.1016/j.chom.2013.07.007 23954159 PMC3772512

[35] Rubinstein MR, Wang X, Liu W, Hao Y, Cai G, Han YW. 2013. Fusobacterium nucleatum promotes colorectal carcinogenesis by modulating E-cadherin/β-catenin signaling via its FadA adhesin. Cell Host Microbe 14:195–206. doi:10.1016/j.chom.2013.07.012 23954158 PMC3770529

[36] Shang F-M, Liu H-L. 2018. Fusobacterium nucleatum and colorectal cancer: a review. World J Gastrointest Oncol 10:71–81. doi:10.4251/wjgo.v10.i3.71 29564037 PMC5852398

[37] Yu TC, Guo F, Yu Y, Sun T, Ma D, Han J, Qian Y, Kryczek I, Sun D, Nagarsheth N, Chen Y, Chen H, Hong J, Zou W, Fang JY. 2017. Fusobacterium nucleatum promotes chemoresistance to colorectal cancer by modulating autophagy. Cell 170:548–563. doi:10.1016/j.cell.2017.07.008 28753429 PMC5767127

[38] Ma C-T, Luo H-S, Gao F, Tang Q-C, Chen W. 2018. Fusobacterium nucleatum promotes the progression of colorectal cancer by interacting with E-cadherin. Oncol Lett 16:2606–2612. doi:10.3892/ol.2018.8947 30013655 PMC6036566

[39] Lawrence GW, McCarthy N, Walsh CJ, Kunyoshi TM, Lawton EM, O’Connor PM, Begley M, Cotter PD, Guinane CM. 2022. Effect of a bacteriocin-producing Streptococcus salivarius on the pathogen Fusobacterium nucleatum in a model of the human distal colon. Gut Microbes 14:2100203. doi:10.1080/19490976.2022.2100203 35877697 PMC9318236

[40] Jain C, Rodriguez-R LM, Phillippy AM, Konstantinidis KT, Aluru S. 2018. High throughput ANI analysis of 90K prokaryotic genomes reveals clear species boundaries. Nat Commun 9:5114. doi:10.1038/s41467-018-07641-9 30504855 PMC6269478

[41] O’Sullivan JN, O’Connor PM, Rea MC, O’Sullivan O, Walsh CJ, Healy B, Mathur H, Field D, Hill C, Paul Ross R. 2020. Nisin J, a novel natural nisin variant, is produced by Staphylococcus capitis sourced from the human skin microbiota. J Bacteriol 202:e00639–19. 31740495 10.1128/JB.00639-19 PMC6964739

[42] Zhang Q, Yu Y, Vélasquez JE, van der Donk WA. 2012. Evolution of lanthipeptide synthetases. Proc Natl Acad Sci USA 109:18361–18366. doi:10.1073/pnas.1210393109 23071302 PMC3494888

[43] Hatziioanou D, Gherghisan-Filip C, Saalbach G, Horn N, Wegmann U, Duncan SH, Flint HJ, Mayer MJ, Narbad A. 2017. Discovery of a novel lantibiotic nisin O from Blautia obeum A2-162, isolated from the human gastrointestinal tract. Microbiology (United Kingdom) 163:1292–1305. doi:10.1099/mic.0.000515 PMC5882112 28857034

[44] Zendo T, Ohashi C, Maeno S, Piao X, Salminen S, Sonomoto K, Endo A. 2020. Kunkecin A, a new nisin variant bacteriocin produced by the fructophilic lactic acid bacterium, Apilactobacillus kunkeei FF30-6 isolated from honey bees. Front Microbiol 11:571903. doi:10.3389/fmicb.2020.571903 33042078 PMC7525160

[45] Garcia-Gutierrez E, Mayer MJ, Cotter PD, Narbad A. 2019. Gut microbiota as a source of novel antimicrobials. Gut Microbes 10:1–21. doi:10.1080/19490976.2018.1455790 29584555 PMC6363078

[46] Oscáriz JC, Pisabarro AG. 2001. Classification and mode of action of membrane-active bacteriocins produced by Gram-positive bacteria. Int Microbiol 4:13–19. doi:10.1007/s101230100003 11770815

[47] Meade E, Slattery MA, Garvey M. 2020. Bacteriocins, potent antimicrobial peptides and the fight against multi drug resistant species: resistance is futile? Antibiotics (Basel) 9:32. doi:10.3390/antibiotics9010032 31963311 PMC7168330

[48] Kaletta C, Entian KD. 1989. Nisin, a peptide antibiotic: cloning and sequencing of the nisA gene and posttranslational processing of its peptide product. J Bacteriol 171:1597–1601. doi:10.1128/jb.171.3.1597-1601.1989 2493449 PMC209786

[49] Bahey-El-Din M, Griffin BT, Gahan CGM. 2008. Nisin inducible production of listeriolysin O in Lactococcus lactis NZ9000.. Microb Cell Fact 7:24. doi:10.1186/1475-2859-7-24 18664263 PMC2515284

[50] Lagedroste M, Reiners J, Smits SHJ, Schmitt L. 2020. Impact of the nisin modification machinery on the transport kinetics of NisT. Sci Rep 10:12295. doi:10.1038/s41598-020-69225-2 32703992 PMC7378552

[51] Renye JA, Somkuti GA, Qi PX, Steinberg DH, McAnulty MJ, Miller AL, Guron GKP, Oest AM. 2024. BlpU is a broad-spectrum bacteriocin in Streptococcus thermophilus. Front Microbiol 15:1409359. doi:10.3389/fmicb.2024.1409359 39081891 PMC11286413

[52] Shin JM, Ateia I, Paulus JR, Liu H, Fenno JC, Rickard AH, Kapila YL. 2015. Antimicrobial nisin acts against saliva derived multi-species biofilms without cytotoxicity to human oral cells. Front Microbiol 6:617. doi:10.3389/fmicb.2015.00617 26150809 PMC4471743

[53] Enigk K, Jentsch H, Rodloff AC, Eschrich K, Stingu C-S. 2020. Activity of five antimicrobial peptides against periodontal as well as non-periodontal pathogenic strains. J Oral Microbiol 12:1829405. doi:10.1080/20002297.2020.1829405 33133417 PMC7580719

[54] Cotter PD, Ross RP, Hill C. 2013. Bacteriocins - a viable alternative to antibiotics? Nat Rev Microbiol 11:95–105. doi:10.1038/nrmicro2937 23268227

[55] Sambola A, Miro JM, Tornos MP, Almirante B, Moreno-Torrico A, Gurgui M, Martinez E, Del Rio A, Azqueta M, Marco F, Gatell JM. 2002. Streptococcus agalactiae infective endocarditis: analysis of 30 cases and review of the literature, 1962-1998. Clin Infect Dis 34:1576–1584. doi:10.1086/340538 12032892

[56] Field D, Connor PMO, Cotter PD, Hill C, Ross RP. 2008. The generation of nisin variants with enhanced activity against specific Gram-positive pathogens. Mol Microbiol 69:218–230. doi:10.1111/j.1365-2958.2008.06279.x 18485077

[57] O’Donnell MM, Rea MC, O’Sullivan Ó, Flynn C, Jones B, McQuaid A, Shanahan F, Ross RP. 2016. Preparation of a standardised faecal slurry for ex-vivo microbiota studies which reduces inter-individual donor bias. J Microbiol Methods 129:109–116. doi:10.1016/j.mimet.2016.08.002 27498348

[58] Kim S, Covington A, Pamer EG. 2017. The intestinal microbiota: antibiotics, colonization resistance, and enteric pathogens. Immunol Rev 279:90–105. doi:10.1111/imr.12563 28856737 PMC6026851

[59] O’Donnell MM, Rea MC, Shanahan F, Ross RP. 2018. The use of a mini-bioreactor fermentation system as a reproducible, high-throughput ex vivo batch model of the distal colon. Front Microbiol 9:1844. doi:10.3389/fmicb.2018.01844 30147684 PMC6096000

[60] Field D, Fernandez de Ullivarri M, Ross RP, Hill C. 2023. After a century of nisin research - where are we now? FEMS Microbiol Rev 47:fuad023. doi:10.1093/femsre/fuad023 37300874 PMC10257480

[61] Jozala AF, Lopes AM, Mazzola PG, Magalhães PO, Vessoni Penna TC, Pessoa A. 2008. Liquid-liquid extraction of commercial and biosynthesized nisin by aqueous two-phase micellar systems. Enzyme Microb Technol 42:107–112. doi:10.1016/j.enzmictec.2007.08.005 22578859

[62] Tagg JR, Dajani AS, Wannamaker LW. 1976. Bacteriocins of Gram-positive bacteria. Bacteriol Rev 40:722–756. doi:10.1128/br.40.3.722-756.1976 791239 PMC413978

[63] 2020. Cutadapt removes adapter sequences from high-throughput sequencing reads | Martin | EMBnet.Journal. Available from: https://journal.embnet.org/index.php/embnetjournal/article/view/200/479. Retrieved 13 Feb 2020.

[64] Andrews S. 2010. FastQC A quality control tool for high throughput sequence data

[65] Bankevich A, Nurk S, Antipov D, Gurevich AA, Dvorkin M, Kulikov AS, Lesin VM, Nikolenko SI, Pham S, Prjibelski AD, Pyshkin AV, Sirotkin AV, Vyahhi N, Tesler G, Alekseyev MA, Pevzner PA. 2012. SPAdes: a new genome assembly algorithm and its applications to single-cell sequencing. J Comput Biol 19:455–477. doi:10.1089/cmb.2012.0021 22506599 PMC3342519

[66] Seemann T. 2014. Prokka: rapid prokaryotic genome annotation. Bioinformatics 30:2068–2069. doi:10.1093/bioinformatics/btu153 24642063

[67] Lagesen K, Hallin P, Rødland EA, Staerfeldt H-H, Rognes T, Ussery DW. 2007. RNAmmer: consistent and rapid annotation of ribosomal RNA genes. Nucleic Acids Res 35:3100–3108. doi:10.1093/nar/gkm160 17452365 PMC1888812

[68] van Heel AJ, de Jong A, Montalbán-López M, Kok J, Kuipers OP. 2013. BAGEL3: automated identification of genes encoding bacteriocins and (non-)bactericidal posttranslationally modified peptides. Nucleic Acids Res 41:W448–53. doi:10.1093/nar/gkt391 23677608 PMC3692055

[69] Carver T, Berriman M, Tivey A, Patel C, Böhme U, Barrell BG, Parkhill J, Rajandream M-A. 2008. Artemis and ACT: viewing, annotating and comparing sequences stored in a relational database. Bioinformatics 24:2672–2676. doi:10.1093/bioinformatics/btn529 18845581 PMC2606163

[70] Altschul SF, Gish W, Miller W, Myers EW, Lipman DJ. 1990. Basic local alignment search tool. J Mol Biol 215:403–410. doi:10.1016/S0022-2836(05)80360-2 2231712

[71] Thompson JD, Higgins DG, Gibson TJ. 1994. CLUSTAL W: improving the sensitivity of progressive multiple sequence alignment through sequence weighting, position-specific gap penalties and weight matrix choice. Nucl Acids Res 22:4673–4680. doi:10.1093/nar/22.22.4673 7984417 PMC308517

[72] Shi LH, Balakrishnan K, Thiagarajah K, Mohd Ismail NI, Yin OS. 2016. Beneficial properties of probiotics. Trop Life Sci Res 27:73–90. doi:10.21315/tlsr2016.27.2.6 27688852 PMC5031164

[73] Wickham H. 2009. ggplot2. 10.1007/978-0-387-98141-3.

[74] Gu Z. 2022. Complex heatmap visualization. iMeta 1:e43. doi:10.1002/imt2.43 38868715 PMC10989952

[75] Community ecology package. 2022. R package vegan version 2.6-4

[76] Kassambara A. 2023. Pipe-friendly framework for basic statistical tests. R package rstatix version 0.7.2

[77] Douglas GM, Kim S, Langille MGI, Shapiro BJ. 2023. Efficient computation of contributional diversity metrics from microbiome data with FuncDiv. Bioinformatics 39:btac809. doi:10.1093/bioinformatics/btac809 36519836 PMC9825779

