# Supplementary material for: A gut-derived Streptococcus salivarius produces the novel nisin variant designated nisin G and inhibits Fusobacterium nucleatum in a model of the human distal colon microbiome
Source: mBio. 2024 Dec 18;16(2):e01573-24. doi: 10.1128/mbio.01573-24 (PMC11796361; doi:10.1128/mbio.01573-24)
Supplement: Supplemental material — Tables S1-S4. [file mbio.01573-24-s0001.docx]

**SUPPLEMENTARY MATERIAL**

**A gut-derived *Streptococcus salivarius* produces the novel nisin variant designated nisin G and inhibits *Fusobacterium nucleatum* in a model of the human distal colon microbiome**

^a^Garreth W. Lawrence, ^b^Enriqueta Garcia-Gutierrez, ^b,c^ A. Kate O’Mahony, ^c^Calum J. Walsh, ^b,c^Paula M. O’Connor, ^a^Máire Begley, ^a*^Caitriona M. Guinane and ^b,c,d*^ Paul D. Cotter

^a^Department of Biological Sciences, Munster Technological University, Cork, T12 P928, Ireland.

^b^Teagasc Food Research Centre, Moorepark, Fermoy, Cork, P61 C996, Ireland.

^c^APC Microbiome Ireland, Cork, T12 YN60, Ireland.

^d^VistaMilk SFI Research Centre, Moorepark, Fermoy, Cork, P61 C996, Ireland.

*authors for correspondence: [paul.cotter@teagasc.ie](mailto:paul.cotter@teagasc.ie); caitriona.guinane@mtu.ie

**Supplementary Table 1. Bacterial strains and their culture conditions used in this study**

| **Indicator organism** | **Culture Medium** | **Temp (°C) + Conditions** |
| --- | --- | --- |
| *Fusobacterium nucleatum* DSM15643 | FAA | 37, Anaerobic |
| *Fusobacterium nucleatum* DSM19507 | FAA | 37, Anaerobic |
| *Fusobacterium nucleatum subsp. vincentii* DSM19508 | FAA | 37, Anaerobic |
| *Fusobacterium periodonticum* DSM19545 | FAA | 37, Anaerobic |
| *Escherichia coli* K12 | LB | 37, Aerobic |
| *Escherichia coli* ATCC25927 | LB | 37, Aerobic |
| *Clostridioides difficile* DPC6357 | FAA | 37, Anaerobic |
| *Streptococcus agalactiae* DPC7040 | BHI | 37, Anaerobic |
| *Streptococcus uberis* DPC4344 | BHI | 37, Anaerobic |
| *Streptococcus mutans* DPC6160 | BHI | 37, Anaerobic |
| *Streptococcus mutans* DPC6161 | BHI | 37, Anaerobic |
| *Streptococcus thermophilus* DPC5472 | BHI | 37, Anaerobic |
| *Streptococcus thermophilus* DPC5657 | BHI | 37, Anaerobic |
| *Streptococcus agalactiae* ATCC13813 | BHI | 37, Anaerobic |
| *Streptococcus simulans* APC3482 | BHI | 37, Anaerobic |
| *Listeria monocytogenes* DPC3564B | BHI | 37, Aerobic |
| *Listeria monocytogenes* DPC3853B | BHI | 37, Aerobic |
| *Listeria innocua* DPC3572 | BHI | 37, Aerobic |
| *Limosilactobacillus fermentum* DPC3320 | BHI | 37, Aerobic |
| *Lactiplantibacillus plantarum* DPC6667 | BHI | 37, Aerobic |
| *Lactobacillus delbrueckii ssp. bulgaricus* DPC5383 | MRS | 37, Anaerobic |
| *Lactococcus lactis* NZ9700 | GM17 | 30, Aerobic |
| *Staphylococcus aureus* DPC7016 | BHI | 37, Aerobic |
| Methicillin-resistant *Staphylococcus aureus* DPC5654 | BHI | 37, Aerobic |
| *Staphylococcus epidermidis* DPC5990 | BHI | 37, Aerobic |

FAA, Fastidious Anaerobic Agar (Lab M, Lancashire, UK); BHI, Brain Heart Infusion; GM17, Glucose (0.5%) M17; (Difco Laboratories, Detroit, MI); LB, Luria-Bertani Medium; MRS, de Man, Rogosa, and Sharpe medium (Difco Laboratories, Detroit, MI); DSMZ, German Collection of Microorganisms and Cell Culture GmbH; ATCC, American Type Culture Collection; APC, Alimentary Pharmabiotic Centre; DPC, Teagasc Culture Collection.

| **Colon model**  **treatment** | **Fermentation**  **timepoint**  **(hours)** | ***F. nucleatum* copy**  **number/μl DNA**  **(mean ± SD)** | **Cycle threshold (Ct)**  **(mean ± SD)** |
| --- | --- | --- | --- |
| *S. salivarius* DPC6487 *+*  *F. nucleatum* DSM15643 | 0 | 4452.8±1547.2 | 20.3±0.4 |
|  | 6 | 9243.3±3408.4 | 22.7±0.4 |
|  | 24 | 139.4±39.7 | 22.4±0.1 |
| *S. salivarius* DPC6487 | 0 | 13.8± 6.7 | 32.7±1.0 |
|  | 6 | 8.8±5.8 | 33.5±1.0 |
|  | 24 | 7.7±3.5 | 33.6±0.9 |
| *F. nucleatum* DSM15643 | 0 | 20797.8±4214.3 | 20.4±0.2 |
|  | 6 | 5071.5±1197.4 | 20.0±0.3 |
|  | 24 | 6237.0± 449.0 | 20.0±0.3 |
| Control | 0 | 7.3±2.0 | 33.6±0.5 |
|  | 6 | 8.3±6.8 | 33.7±1.4 |
|  | 24 | 6.8±2.7 | 33.7±0.6 |

**Supplementary Table 2. Real time-quantitative polymerase chain reaction (RT-qPCR) quantification of *F. nucleatum* in colon model wells**

**Supplementary Table 3. Statistical testing of differences between *S. salivarius* DPC6487-treated and untreated samples after 24 hours fermentation (n = 6 in each group).**

| **Taxonomic Data** | | | | | | |
| --- | --- | --- | --- | --- | --- | --- |
| **Data** | **Diversity** | **Metric** | **Method** | ***p* value** | **F (Test Statistic)** | **sig** |
| MetaPhlAn4 | α | Richness | Kruskal-Wallis rank sum | 0.2290 | 1.446935 |  |
|  |  | Shannon | Kruskal-Wallis rank sum | 0.6809 | 0.169169 |  |
|  |  | Inverse Simpson | Kruskal-Wallis rank sum | 0.6578 | 0.196196 |  |
|  |  | Berger-Parker (dominance) | Kruskal-Wallis rank sum | 0.0104 | 6.564103 | * |
|  |  | Gini (dominance) | Kruskal-Wallis rank sum | 0.5218 | 0.410256 |  |
|  | β | Bray-Curtis dissimilarity | PERMANOVA (adonis2) | 0.0019 | 14.56514 | ** |
|  |  | Robust Aitchison distances | PERMANOVA (adonis2) | 0.5082 | 0.869299 |  |
|  |  | Weighted UNIFRAC distances | PERMANOVA (adonis2) | 0.0023 | 33.70637 | ** |
|  |  | Unweighted UNIFRAC distances | PERMANOVA (adonis2) | 0.5222 | 0.659969 |  |
| **Functional Data** | | | | | | |
| SUPER-FOCUS Level 1 | β | Robust Aitchison distances | PERMANOVA (adonis2) | 0.0927 | 1.54098323 | . |
| SUPER-FOCUS Level 2 |  | Robust Aitchison distances | PERMANOVA (adonis2) | 0.0037 | 3.05205156 | ** |
| SUPER-FOCUS Level 3 |  | Robust Aitchison distances | PERMANOVA (adonis2) | 0.0019 | 2.87231462 | ** |
| HUMAnN4 (Stratified) |  | Robust Aitchison distances | PERMANOVA (adonis2) | 0.1311 | 1.38408 |  |
| HUMAnN4 (Unstratified) |  | Robust Aitchison distances | PERMANOVA (adonis2) | 0.3086 | 1.062284 |  |

**Supplementary Table 4. Statistically significant associations with *S. salivarius* DPC6487-treatment for (A) species (MetaPhlAn4) and (B) functional pathways (HUMAnN4), after 24 hours fermentation. Pathways were filtered to remove associations with pathways mapped at genus-level to *Streptococcus* (n = 198).** Sign of the MaAsLin2 coefficient (coef) indicates the directionality of the association (towards any *S. salivarius* DPC6487 treatment), while the magnitude approximates the effect size. MaAsLin2 q-value (qval) denotes respective p-value after adjustment for multiple testing (Benjamini-Hochberg).

| **Species** | **coef** | **qval** |
| --- | --- | --- |
| Streptococcus_salivarius | 3.697978 | 8.63E-08 |
| Bifidobacterium_longum | -0.58126 | 0.00238 |
| Bifidobacterium_animalis | -0.56784 | 0.008937 |
| Lactobacillus_acidophilus | -0.89439 | 0.048063 |
| **Pathways** | **coef** | **qval** |
| PWY.6609..adenine.and.adenosine.salvage.III.g__Escherichia.s__Escherichia_coli | -2.92243 | 0.000991 |
| PWY0.1296..purine.ribonucleosides.degradation.g__Escherichia.s__Escherichia_coli | -2.32189 | 0.037757 |
| PWY.7234..inosine.5..phosphate.biosynthesis.III.unclassified | -1.43094 | 0.003992 |
| PWY.5941..glycogen.degradation.II.g__Bifidobacterium.s__Bifidobacterium_longum | -0.547 | 0.027621 |
| UNINTEGRATED.g__Bifidobacterium.s__Bifidobacterium_longum | -0.50253 | 0.022517 |
| PWY.7221..guanosine.ribonucleotides.de.novo.biosynthesis.g__Bifidobacterium.s__Bifidobacterium_catenulatum | 1.135627 | 0.013414 |
| PWY.7851..coenzyme.A.biosynthesis.II..eukaryotic..g__Alistipes.s__Alistipes_putredinis | 1.203684 | 0.003231 |
| P108.PWY..pyruvate.fermentation.to.propanoate.I.unclassified | 1.219807 | 0.023099 |
| COBALSYN.PWY..superpathway.of.adenosylcobalamin.salvage.from.cobinamide.I.g__Faecalibacterium.s__Faecalibacterium_prausnitzii | 1.403665 | 0.002981 |
